# BACE1 and SCD1 are associated with neurodegeneration

**DOI:** 10.3389/fnagi.2023.1194203

**Published:** 2023-09-08

**Authors:** Ferley A. Bedoya-Guzmán, Mar Pacheco-Herrero, Ivan Daniel Salomon-Cruz, Angela Maria Barrera-Sandoval, Johanna Andrea Gutierrez Vargas, Javier Gustavo Villamil-Ortiz, Carlos Andres Villegas Lanau, Julián David Arias-Londoño, Estela Area-Gomez, Gloria Patricia Cardona Gomez

**Affiliations:** ^1^Faculty of Medicine University of Antioquia, Cellular and Molecular Neurobiology Area and Neurobank, Group of Neuroscience (GNA), Medellín, Colombia; ^2^Neuroscience Research Laboratory, Faculty of Health Sciences, Pontificia Universidad Católica Madre y Maestra, Santiago de los Caballeros, Dominican Republic; ^3^Grupo de Investigación en Salud del Adulto Mayor (GISAM), Corporación Universitaria Remington, Medellín, Colombia; ^4^Department of Systems Engineering, University of Antioquia UdeA, Medellín, Colombia; ^5^Department of Neurology, Columbia University Medical Center, New York, NY, United States

**Keywords:** BACE1, SCD1, neurodegeneration, phospholipids, PUFAs, pro-inflammation

## Abstract

**Introduction:**

Proteolytic processing of amyloid protein precursor by β-site secretase enzyme (BACE1) is dependent on the cellular lipid composition and is affected by endomembrane trafficking in dementia and Alzheimer's disease (AD). Stearoyl-CoA desaturase 1 (SCD1) is responsible for the synthesis of fatty acid monounsaturation (MUFAs), whose accumulation is strongly associated with cognitive dysfunction.

**Methods:**

In this study, we analyzed the relationship between BACE1 and SCD1 *in vivo* and *in vitro* neurodegenerative models and their association in familial AD (FAD), sporadic AD (SAD), and cerebral autosomal dominant arteriopathy with subcortical infarcts and leukoencephalopathy (CADASIL) using microscopy, biochemical, and mass SPECT approach.

**Results:**

Our findings showed that BACE1 and SCD1 immunoreactivities were increased and colocalized in astrocytes of the hippocampus in a rat model of global cerebral ischemia (2-VO). A synergistic effect of double BACE1/SCD1 silencing on the recovery of motor and cognitive functions was obtained. This neuroprotective regulation involved the segregation of phospholipids (PLs) associated with polyunsaturated fatty acids in the hippocampus, cerebrospinal fluid, and serum. The double silencing in the sham and ischemic groups was stronger in the serum, inducing an inverse ratio between total phosphatydilcholine (PC) and lysophosphatidylcholine (LPC), represented mainly by the reduction of PC 38:4 and PC 36:4 and an increase in LPC 16:0 and LPC 18:0. Furthermore, PC 38:4 and PC:36:4 levels augmented in pathological conditions in *in vitro* AD models. BACE1 and SCD1 increases were confirmed in the hippocampus of FAD, SAD, and CADASIL.

**Conclusion:**

Therefore, the findings suggest a novel convergence of BACE-1 and SCD1 in neurodegeneration, related to pro-inflammatory phospholipids.

## Introduction

Alzheimer's disease (AD) is a multifactorial neurodegenerative disorder that results in the progressive loss of memory and other emotional and cognitive dysfunctions. AD brains present with two main hallmarks, namely, extracellular neuritic plaques mainly composed of amyloid β (Aβ) fibrils and neurofibrillary tangles formed by hyperphosphorylated tau protein (Hardy and Selokoe, 2002). Aβ, a key pathogenic structure in AD (Viola and Klein, 2015), is derived from the amyloid precursor protein through a sequential cleavage by the β-site secretase enzyme (BACE1) to form the C-terminal fragment of 99-aa (C99), which is processed by γ-secretase (Sun and Roy, 2018). The levels and activity of BACE1, which is the rate-limiting step in Aβ production (Sun and Roy, 2018), are higher in the AD brain (Fukumoto, 2002; Yang et al., 2003). BACE1 is a type I membrane-bound aspartyl protease (Sathya et al., 2012; Yuksel and Tacal, 2019) that was initially synthesized as a zymogen (pro-BACE) in the endoplasmic reticulum (ER) (Cole and Vassar, 2007). BACE1 is highly expressed in neurons and secondarily in astrocytes and oligodendrocytes (Zhao et al., 1996; Rossner et al., 2005).

BACE1 activity is modulated by post-translational modifications, such as palmitoylation or thioesterification with 16-carbon saturated fatty acids (SFAs) (palmitate, C16:0), which have been shown to contribute to Aβ production in the brain (Cho and Park, 2016; Yuksel and Tacal, 2019). In fact, it has been shown that a palmitate-enriched diet increases BACE1 activity and Aβ generation in neuroblastoma cells and mice through different transcription factors (Marwarha et al., 2019). In addition, some phospholipids, such as phosphatidylethanolamine (PE), mainly esterified with SFAs, modify the anchoring in lipid rafts of BACE1 bound to glycosylphosphatidylinositol (Sambamurti et al., 1999).

Genome-wide association studies have highlighted the role of lipid alterations in the cell as critical components of AD pathogenesis (Kunkle et al., 2019). Our previous studies in animal models of cognitive impairment (ischemic stroke, 3xTg-AD mice) and human brains with dementia [Cerebral autosomal dominant arteriopathy with subcortical infarcts and leukoencephalopathy (CADASIL), familial AD (FAD), and sporadic AD (SAD)] revealed common disturbances in phospholipid (PL) unsaturation, mainly producing phospholipids (PLs) associated with arachidonic derivatives and monounsaturated fatty acids (MUFAs) (Villamil-Ortiz et al., 2016; Sabogal-Guáqueta et al., 2018, 2020). In agreement, previous studies have highlighted the association between higher levels of MUFAs and an increased risk of developing AD (Morris et al., 2003; Astarita et al., 2011). The expression of stearoyl-CoA desaturase 1 (SCD1), the rate-limiting enzyme in the synthesis of oleate (18:1n-9) and palmitoleate (16:1n-7), respectively, from the SFAs stearate (18:0) and palmitate (16:0) is consistent with this (Peck and Schulze, 2016). It has been described that SCD1 expression is highest in the central and peripheral nervous systems after injury (Lengi and Corl, 2015) and in pathological conditions such as AD (Corpeleijn et al., 2006; Warensjö et al., 2007; Mar-Heyming et al., 2008; Astarita et al., 2011). Also, Nuber et al. (2021) showed how the inhibition of SCD1 activity for *in vitro* and *in vivo* models of Parkinson disease was able to decrease the severe neuropathology by ameliorating dopaminergic fiber loss and the consequent motor decline.

It is known that SCD1 plays a crucial role in cellular membrane physiology and signaling (Peláez et al., 2020), inflammation (Ralston et al., 2016; Liu et al., 2017), and metabolism. Defects in SCD1 function lead to metabolic syndrome, insulin resistance, cancer, and neurological disorders (Igal, 2016; Ravaut et al., 2020). In addition, we found that BACE1 depletion results in lipidomic alterations, specifically changes in PL monounsaturation and polyunsaturation in the hippocampus of 3xTgAD mice, correlating with improved cognitive function and a significant reduction of taupathy (Piedrahita et al., 2016; Villamil-Ortiz et al., 2016). Notably, the neuroprotective effect of BACE1's silencing was SCD1-dependent (Villamil-Ortiz et al., 2018). Moreover, β-amyloidosis induces the specific upregulation of SCD1 in macrophages (Uryu et al., 2003), which in turn induces an alteration in the reparative properties of macrophages and microglia (Bogie et al., 2020). Although BACE1 and SCD1 have been independently involved in myelinization and axonal growth (Truong et al., 2019; Bogie et al., 2020), the potential relationship between the effects of these proteins and their contribution to the lipid disturbances observed in the context of cognitive impairment has not been characterized yet, which is the focus of the work presented here.

## Materials and methods

### Animal procedures

All animal procedures were performed in accordance with the ARRIVE guidelines, the Guide for the Care and Use of Laboratory Animals, 8th edition, published by the NIH, and the Colombian standards (law 84/1989 and resolution 8430/1993). These procedures were approved by the Ethics Committee for Animal Experimentation of the University of Antioquia, Medellin, Colombia.

Male Wistar albino rats from our in-house, pathogen-free colony in the vivarium at SIU (Sede de Investigación Universitaria), University of Antioquia, Medellin, Colombia, were kept on a 12:12 h dark/light cycle and received food and water *ad libitum*. Special care was taken to minimize animal suffering and reduce the number of animals used. Three-month-old rats weighing 380–450 g were used in this study (Cardona-Gomez et al., 2004; Martins and Creegan, 2014).

### Global cerebral ischemia (2-VO)

A week later, after the short hairpin BACE1 RNAmiR delivery, the animals were anesthetized using ketamine (60 mg/kg) and xylazine (5 mg/kg) for initial deep sedation, and for keeping more than 30 min in deep surgical plane, animals received a 2%−4% isoflurane and 96% oxygen mixture *via* an inhalation anesthesia machine. A variation in the global cerebral ischemic model was implemented, involving a 2-vessel occlusion (2-VO) (Marosi et al., 2006). The right common carotid artery (CCA) was permanently occluded using a 6.0-gauge nylon suture (Corpaul, Bogota, Colombia), and the left CCA was obstructed for 20 min using a vascular clip. After 20 min, the vascular clip was removed to allow reperfusion. The sham control rats underwent the same procedure without CCA occlusion.

### ShBACE1miR and shSCD1 deliveries *in vivo*

The animals were anesthetized (5% ketamine and 2% xylazine, 50:5 dosage mg/kg) and injected with 1 μl of AAV2-shSCRmiR (shSCRmiR) or AAV2-shBACE1miR (shBACE1miR) into the right hippocampus (Bregma coordinates were as follows: 2.52 mm anteroposterior, 0.8 mm right, and 3.6 mm dorsoventral). The injections were performed with a 10-μl syringe (Hamilton, Reno, NV, USA) at 0.1 μl/min, and 10 min elapsed after the infusion before the syringe was withdrawn. Additional stereotaxic injection of short hairpin SCD1 (Santa Cruz Biotechnology) was performed 30 min after global cerebral ischemia. The animals were injected with 2 μl of lentiviruses shSCR (shSCR) or lenti-shSCD1 (shSCD1) into the right hippocampus (bregma coordinates were −2.52 mm anteroposterior, 0.8 mm right, and 3.6 mm dorsoventral). The injections were performed with a 10-μl syringe (Hamilton, Reno, NV, USA) at 0.1 μl/min, and 10 min elapsed after the infusion before the syringe was withdrawn. The animals were sacrificed a month post-ischemia for lipid analyses after the behavioral tests.

### Morris water maze

The animals were evaluated using the Morris water maze 2 weeks after shBACEmiR injection and one week after ischemia from 2VO and shSCD1 were injected. The behavioral test consisted of a black plastic tank filled with water (22 ± 2°C), where a hidden platform (12 cm diameter) was submerged 3 cm below the water level during spatial learning, memory, and relearning tests and 1.5 cm above the surface of the water during the visible session. Extremized visual cues around the room remained fixed throughout the experiment. Six sessions, or trials, were performed for the learning evaluation. Each session consisted of four successive sub-trials (90 s per trial and 30 s intertrial intervals), and each sub-trial began with the rat being placed pseudo-randomly in one of four starting locations (N, S, W, and E). The animals were then provided with a 48-h retention period, followed by a probe trial of spatial reference memory in which the animals were placed in the tank without the platform for 90 s. The latency to reach the exact former location of the platform was recorded during the probe trial. Subsequently, the platform was moved to a new location. Each animal's ability to learn the new location was measured by determining the latency in four sessions conducted in the same manner as the learning phase. The latency to reach the platform was evaluated using a visible platform to control for any differences in visual-motor abilities or motivation between the experimental groups. The behavior of the animals was recorded using an automated system (Viewpoint, Lyon, France).

### Neurological evaluation

Neurological performance was evaluated 6 h after cerebral ischemic stroke. Neurological function was scored on an 18-point scale based on the Garcia test (Garcia et al., 1995). The neurological score was based on the following six different neurological tests: (1) spontaneous activity; (2) symmetry in limb movement; (3) forepaw outstretching; (4) climbing; (5) body proprioception; and (6) response to vibrissae touch. Each test was scored with a maximum of three points based on a set of predetermined criteria, as described by Garcia et al. The scores of each test were summed; the highest possible score was 18 points, indicating no neurological deficits, and the lowest possible score was three points, indicating severe impairment. Neurological assessments were performed daily in the same order.

### Inclined plane test

After 6 h of the 2VO surgery and during the following 7 days, each animal's ability to maintain postural stability was assessed using the inclined plane test. Rats were placed on a smooth wood plate covered with a rubber pad, and the inclined plate was placed. The body axis is parallel to the vertical axis of the plate. The plate angle was increased by 5° in each trial. The relative angle at which the rat could no longer maintain its position for 10 s was recorded as the final score, and it was considered a measure of functional impairment.

### Western blotting

Cerebral cortex was homogenized in lysis buffer (150 mM NaCl, 20 mM Tris (pH 7.4), 10% glycerol, 1 mM EDTA, 1% NP40, and 1 mg/ml inhibiting cocktail proteases). Sodium dodecyl sulfate–polyacrylamide gel electrophoresis (12% SDS–PAGE) was performed using a mini-protein system (Bio-Rad) with low-range molecular-weight standards (Bio-Rad). Protein (30 μg) was loaded into each lane with a loading buffer containing 0.375 M Tris (pH 6.8), 50% glycerol, 10% SDS, 0.5 M DTT, and 0.002% bromophenol blue. Samples were heated at 95°C for 3 min prior to gel loading. After electrophoresis, the proteins were transferred to nitrocellulose membranes (Amersham, Buckinghamshire, United Kingdom) using an electrophoretic transfer system (mini-Trans-Blot Electrophoretic Transfer Cell) at 250 mA for 2 h. The membranes were then washed with TTBS (20 mM Tris–HCl, pH 7.5; 500 mM NaCl; 0.05% Tween-20 containing Tris-buffered saline solution, pH 7.4) and 5% nonfat dry milk and incubated overnight at 4°C with the following primary antibodies: anti-mouse SCD1 (E8 1:500; Santa Cruz Biotechnology, Santa Cruz, CA, United States) and anti-mouse actin (1:1,000; Sigma-Aldrich), which were used as loading controls. Li-COR Biosciences images of fluorescent blots were analyzed using the Odyssey Infrared Imaging System application software version 3.0 (Li-COR). The results from each membrane were normalized to control values. Samples from all experimental groups were processed in parallel to minimize inter-assay variation.

### Lipid analyses

Hippocampus (0.5 g), CSF (0.01 ml), serum (0.5 ml), and *in vitro* cells (1.5 × 105) were processed individually according to the FOLCH technique for the extraction of lipids (Folch et al., 1957) using a mixture of 2 ml of chloroform (CHCl3) and 1 ml of methanol (CH3OH) in a 2:1 (v/v) ratio. Next, 0.005% butylated hydroxytoluene was added, and this mixture was used to homogenize the samples. Subsequently, 1 ml of 0.9% sodium chloride (NaCl) was added, and the mixture was centrifuged at 3,000 rpm for 3 min. The organic layer (lower layer) was removed and transferred to a new glass tube. The solvents were evaporated, and the extract was lyophilized to remove excess humidity. Dry lipids, on average, were 7.1 mg, 0.3 mg, and 2.9 mg for the right hippocampus, CSF, and serum, respectively. And dry lipids from *in vitro* cells were 0.2–0.4 g. Finally, the lipid composition was analyzed by mass spectrometry.

### Mass spectrometry

An automated ESI-MS/MS method was used, and data acquisition and analysis were performed at the Kansas Lipidomics Research Center using an API 4000™ and QTRAP (4000 QTRAP) detection system, as described previously (Zhou et al., 2012). This protocol allowed for the detection and quantification of low concentrations of polar lipid compounds. The molecules were determined by the mass/charge ratios and then compared with their respective internal standards to define which species of lipids were in the evaluated extract: 0.30 nmol of 14:0 LPG, 0.30 nmol of 18:0 LPG, 0.30 nmol of 14:0 PG, 0.30 nmol of 14:0-LPE, 0.30 nmol of 18:0-LPE, 0.60 nmol of 13:0-LPC, 0.60 nmol of 19:0-LPC, 0.60 nmol of 12:0-PC, 0.60 nmol of 24:1-PC, 0.30 nmol of 14:0 LPA, 0.30 nmol of 18:0 LPA, 0.30 nmol of 14:0-PA, 0.30 nmol of 20:0 (phytanoyl)-PA, 0.20 nmol of 14:0-PS, 0.20 nmol of PS, 0.28 nmol of 16:0-18:0 PI, and 0.10 nmol of 18:0-PI. The system identified 13 different lipid species and their respective subspecies, which were recognized by the number of carbons and degree of chain unsaturation. The lipid concentration was normalized to the molar concentration across all species for each sample. The final data are presented as mean % Mol.

### *In vitro* studies and cell lines

Astrocytes and neuronal primary cultures were obtained from the cortices of P1 neonates and E18-19 Wistar rats, respectively. Endothelial cell line bEnd.3 (ATCC CRL-2299) from murine brain microvasculature was used. Also, astrocyte-endothelial cells and astrocyte-neuron cocultures were done to analyze the effect of BACE1 and/or SCD1 inhibition. For details, see Supplementary material and methods.

AD and control cell lines were obtained from the Coriell Institute for Medical Research (Camden, NJ, United States). WT and PSEN1-/-PSEN2- (called PS-DKO) mouse MEFs were provided by Dr. Bart De Strooper (University of Leuven). The purification of mitochondria was performed and analyzed as described (Area-Gomez et al., 2009; Montesinos et al., 2020). Staining of lipid droplets was performed using HCS LipidTox™ Deep Green neutral lipid stain (Invitrogen H34475) according to the manufacturer's instructions. Lipid droplet staining was quantified using ImageJ. PS1-mutant FAD and SAD cells and serum samples were kind gifts from Dr. Gary E. Gibson (Cornell University) to Dr. Estela Area-Gomez (Columbia University).

### Human brain tissue

Postmortem brain tissue from the hippocampus and adjacent entorhinal cortex of five patients with FAD (PS1 mutation E280A), SAD, CADASIL, and healthy controls, obtained from the University of Antioquia's Brain Bank, was included in this study (Table 1). Informed consent for research was obtained from each participant and/or their legal representative, and this study was approved by the Bioethical Committee for Human Studies from the University of Antioquia.

**Table 1:** Cases used in this study.

**Condition**	**Gender**	**Onset Age**	**Age of death**	**CERAD**	**Braak**	**Thal**
Control—Healthy	F	NA	67	0	1	0
Control—Healthy	F	NA	75	0	0	0
Control—Healthy	M	NA	69	A	1	2
Control—Healthy	M	NA	61	0	0	0
Control—Healthy	F	NA	44	0	0	0
FAD (E280A)	F	49	62	B	4	5
FAD (E280A)	F	44	50	C	6	5
FAD (E280A)	F	50	63	C	6	5
FAD (E280A)	M	49	59	C	6	5
FAD (E280A)	F	51	65	B	5	5
Late SAD	F	82	92	C	5	4
Late SAD	F	62	74	B	5	4
Early SAD	F	55	76	B	4	5
SAD	F	81	94	B	4	5
SAD	F	92	98	A	3	3
CADASIL	F	52	65	B	1	2
CADASIL	F	35	45	0	0	0
CADASIL	F	32	49	0	0	0
CADASIL	M	41	59	0	0	0
CADASIL	F	55	78	0	0	0

NA, Non apply.

Tissue blocks were fixed by immersion in a solution of 4% paraformaldehyde in 0.1 M phosphate buffer (PB), pH 7.4, at 4°C for 7 days. Fragments of the hippocampus were frozen at −80°C.

### Immunohistochemistry

Free floating tissue sections (50-μm thick) were permeabilized in 98% formic acid for 5–6 min at 85°C and 30% Triton X-100 in 0.1 M PB for 5 min. Endogenous peroxidase activity was blocked using methanol with 1% hydrogen peroxide (H_2_O_2_) in 0.1 M PB for 20 min at room temperature. Non-specific antibody binding sites were blocked with 1% bovine serum albumin (BSA) (Sigma) and 0.3% Triton X-100 in 0.1 M PB for 1 h. Human sections were then incubated with rabbit BACE1 (ab108394, Abcam, 1:100), mouse SCD1 (ab19862, Abcam, 1:100), mouse anti-human PHF-tau (AT8, MN1020, Thermo, 1:1,000), or phospho-cytosolic phospholipase A2 (p-cPLA2, #2831Cell Signaling, 1:500) primary antibodies. Rat sections were incubated with anti-mouse BACE1 (3C1C3 1:500; ThermoFisher) and anti-SCD1 (E-15, 1:200 Santa Cruz Biotechnology, Santa Cruz, CA, United States), which recognizes the n-terminus of SCD1. All antibodies were diluted with 0.3% Triton X-100 and 0.3% BSA in 0.1 M PB at 4°C for three nights. Slices were then incubated with biotin-labeled mouse and rabbit secondary antibodies (Thermofisher, 1:250) for 1 h and then incubated with the ABC–HRP complex (Thermofisher) for 30 min. Staining was visualized using diaminobenzidine, which was activated by H_2_O_2_. Slices were dehydrated, covered with mounting solution, viewed using a light microscope (Nikon Eclipse E200), and images were captured using a Nikon digital camera (Sight DS-L1) at 4 ×, 10 ×, and 40 × magnification. The images were analyzed using binary thresholding in the ImageJ software (https://imagej.nih.gov/ij/) (National Institutes of Health, NIH) to evaluate the optical density of immunohistochemical staining and represented as percentage (%) with respect to the control in the total area of the hippocampus (CA1 and CA4 areas) and subiculum in human brains and CA2 and Hilux in rat brains.

### Immunofluorescence

For IF staining, we performed a procedure similar to that described for immunohistochemistry. After antigenic retrieval, autofluorescence and background were reduced with 0.1% Sudan Black B in 70% ethanol for 10 min. Sections were incubated with the primary antibody cocktail BACE1 (ab108394, Abcam, 1:100) and SCD1 (ab19862, Abcam, 1:100) or BACE1 and anti-human PHF-tau (AT8, MN1020, Thermo, 1:1,000) in 0.3% Triton X-100 and 0.3% BSA in 0.1 M PB at 4°C for three nights. Rat sections were preincubated for 1 h in 1% BSA containing 0.3% Triton X-100 in 0.1 M P and incubated with primary antibodies in the following combinations: anti-GFAP rabbit (1:500; ab-5804); anti-SCD1 goat (1:500; E15 sc-14720); and anti-BACE1 mouse (1:100; MAI-177 3C1C3) overnight at 4°C in shaking. The secondary antibodies used were anti-rabbit Alexa 488 and anti-mouse 594 fluorescent antibodies (Invitrogen) diluted 1:750 for 1 h in human tissues and anti-mouse Alexa 350, anti-rabbit Alexa 488, and anti-Goat Alexa 594 in rat tissues. All sections incubated in parallel without a primary antibody were included as negative controls for autofluorescence and background binding of secondary antibodies. Sections were visualized using an Olympus IX 81 epifluorescence microscope with a 40x objective (N.A. 0.5). The fluorescence intensity of the triple-labeled images was analyzed using the NIH Image J software (https://imagej.nih.gov/ij/) for rat tissue IF.

### Confocal microscopy analysis

Double immunolabeled sections were mounted in Fluoro-Gel mounting medium (Electron Microscopy Sciences) and imaged using a laser scanning confocal microscope (U-TBi90, Olympus, Japan) using a 60 × dry objective (N.A. 4.2) and the Olympus Fluoview 1000 program. In total, 10 fields in the CA1 area were registered, and 15–20 consecutive single sections were obtained at 0.5-μm intervals in all channels throughout the z-axis of the sample. Image acquisition settings were maintained between the cases. Maximal projection images for every field were generated, and regions of interest (ROIs) were quantified and used to obtain mean gray values from every positive fluorescent signal using the ImageJ software (NIH). The mean gray value for each protein was expressed as the difference between the mean gray value obtained for each ROI and the mean gray value of the corresponding channel's background.

In addition, the images were transformed to 8 bits and subsequently processed and analyzed in the FIJI software. To determine fluorescence intensity, we used a triangle algorithm to segment and threshold our immunostaining images before measuring total and colocalized stained areas. The colocalized signals were obtained using the AND algorithm from the image calculator tool.

### Immunoprecipitation

The hippocampus samples were lysed in 10 mM Tris (pH 7.4), 100 mM NaCl, 1 mM EDTA, 1 mM EGTA, 10% glycerol, 1% NP40, 1 nM orthovanadate, 5 mM NaF, 1 mM phenylmethylsulfonyl fluoride, and a protease inhibitor cocktail (Sigma-Aldrich; Cardona-Gomez et al., 2004). The lysates were clarified by centrifugation at 14,000 rpm for 5 min. A protein assay was performed, and 200 μg of protein was incubated overnight at 4°C in the presence of BACE1 Ab (ab108394, Abcam, 1:100). Protein G-sepharose beads were added, and the samples were incubated for an additional 2 h at RT. The immune complexes were washed three times using immunoprecipitation (IP) lysis buffer before SDS-PAGE and immunoblotting. The proteins were separated using 10% SDS-PAGE, transferred onto nitrocellulose membranes (Amersham), and probed with anti-human PHF-tau (AT8, MN1020, Thermo, 1:250), rabbit cPLA2 (ab198898, Abcam, 1:250), and mouse SCD1 (ab19862, Abcam, 1:250). The lysates were used as the positive control, and incubation with the IgG peptide was used as the negative control for IP.

### Statistical analysis

The statistical analysis of immunostaining was performed using the Minitab program. The normality of the data was assessed using the Kolmogorov-Smirnov test. Normally distributed data are expressed as the mean ± standard error of the mean (SEM). Statistical comparisons were performed using the Student's *t*-test and ANOVA, as noted in the figure legends. The data that were not normally distributed were compared using the Kruskal-Wallis test (more than two groups), followed by Dunnett's *post-hoc* test. Significance was set at a 95% confidence level. Correlations were analyzed using Spearman's Rho test. In addition, to evaluate the interaction between variables, a two-way ANOVA was performed for retention and memory retrieval.

Comparisons among groups were assessed by one-way ANOVA followed by Tukey's *post-hoc* test or the Kruskal-Wallis test, depending on the normal distribution of the experimental data from *in vivo, in vitro*, and post-mortem data. The lipid levels for each sample were calculated by summing the total number of moles of all lipid species measured and then normalizing that total to % Mol. Multivariate analysis was performed using partial least squares-discriminant analysis (PLS-DA) (Ruiz-Perez et al., 2020) to select the correct hyperplane when using few samples and when the separation between the clusters is low (values close to 0). We also used principal components analysis (PCA) (Jung and Marron, 2009; Björklund, 2019; Ruiz-Perez et al., 2020). Analysis by PLS-DA was performed using the routines described by Ballabio and Todeschini (2009) to identify differences among the study groups (Sham and ischemic groups). An index representing the importance of the variables according to the first three components was estimated, which was called the variable importance in projection (VIP) (Mehmood et al., 2012), a weighted sum of the squares of the PLS weight that indicates the importance of each variable in the model and reflects the proportion of the explained variance weighted by the covariance between the predictor variables and the dependent variable (i.e., the groups). For comparison, the recently proposed significance multivariate correlation (sMC) index (Tran et al., 2014) was also included in the analyses. sMC is similar to VIP but discards residual variance in the predictor variables that can be considered non-relevant information to discriminate among the groups. Confidence ellipsoids per group and treatment were also included. Furthermore, PLS-DA was performed iteratively until the smallest subset of variables was identified to obtain separability among the confidence ellipsoids of the groups whenever such separability was observed with the whole set of variables. To evaluate the similarities among the % Mol values of the lipid species, Pearson's linear correlation coefficient was also measured. The data from the univariate statistics were expressed as the mean ± SEM. The statistical significance is indicated in the figures.

## Results

### BACE1 and SCD1 are associated with astrogliosis in a neurodegenerative model

**Figure 1:**
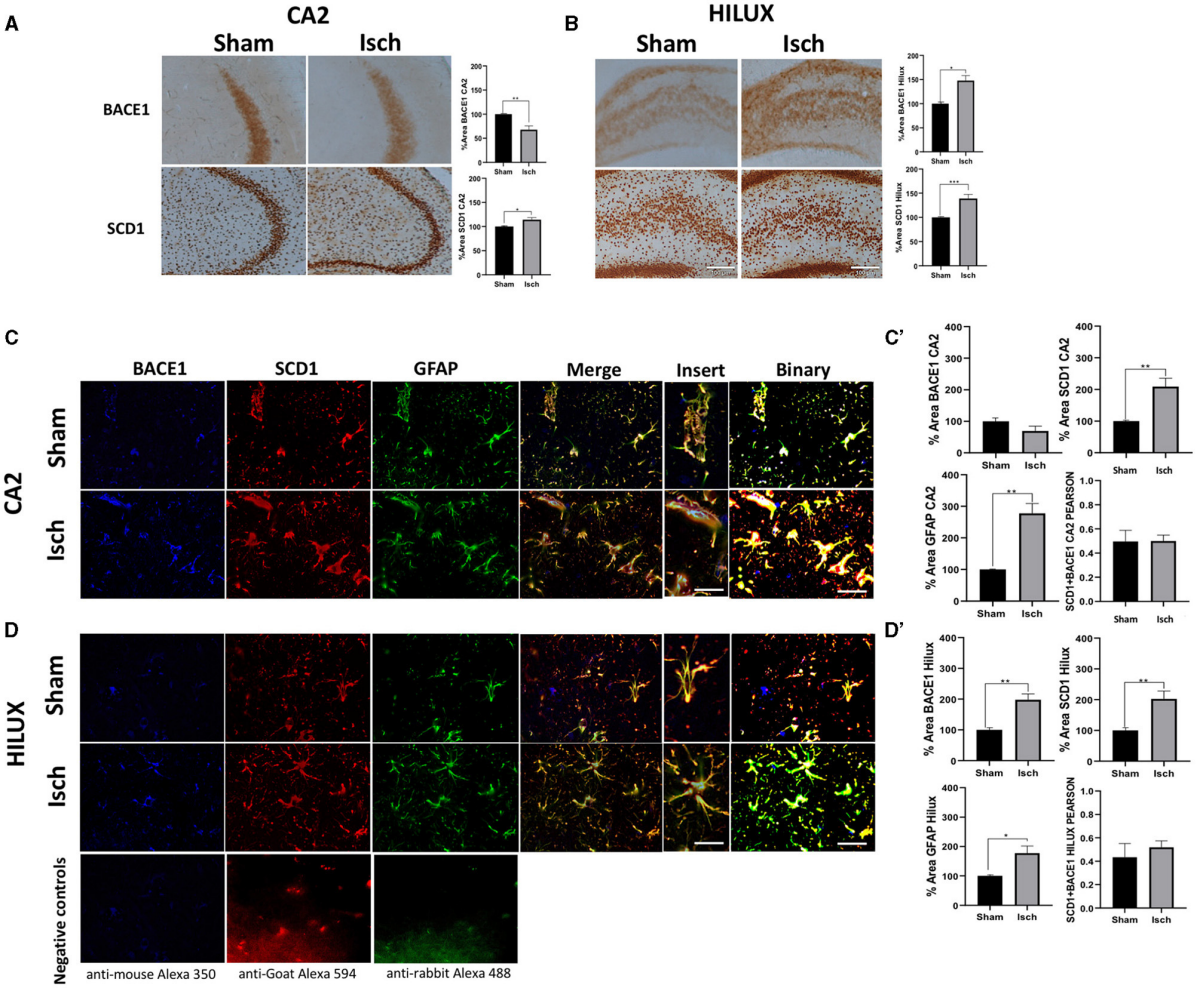
BACE1 and SCD1 colocalize in astrocytes and increase in a neurodegenerative context. **(A)** BACE1 and SCD1 immunoreactivities at the CA2 and **(B)** Hilux from sham and ischemic rats. Representative images 10X, bar graph = 100 nm. The bars indicate the SEM values of the percentage of optical density relative to the control group. **(C)** Representative images of triple fluorescence immunolabeling for BACE1 (blue), SCD1 (red), and GFAP (green) IF at the CA2. **(D)** Hilux from sham and ischemic rats. The merge and colocalization filter (yellow) and binary tool for each condition are shown. Negative controls staining without primary antibodies are shown in **(D)**. Images at 40X, bar graph: 30μm; inset = 60×, bar graph: 10μm. The bars indicate the SEM values. **(C')** %Area for IF intensity of BACE1, SCD1, GFAP, and Pearson correlation of GFAP (BACE1/SCD1) from CA2. **(D')** Hilux. The Student's t-test for comparison between groups was used (*n* = 4, ^*^p ≤ 0.05, ^**^p ≤ 0.001).

Hippocampus from ischemic rats showed increased immunoreactivities for BACE1 and SCD1 proteins in the CA2 and Hilux (Figures 1A, B). Also, there was a general localization of those proteins in astrocytes more than neurons and microglia, whose expressions overlapped and increased concomitantly with the astrogliosis induced by the ischemia (Figures 1C, C', D, D'), which was supported by a significant increase of GFAP, BACE1, and SCD1 IFs and each overlap between them (Figures 1C, C', D, D'), and a similar Pearson correlation in sham and ischemic areas (Figures 1C', D').

### Synergistic effect of BACE and SCD1 silencing on motor and cognitive recovery in an animal model of neurodegeneration

**Figure 2:**
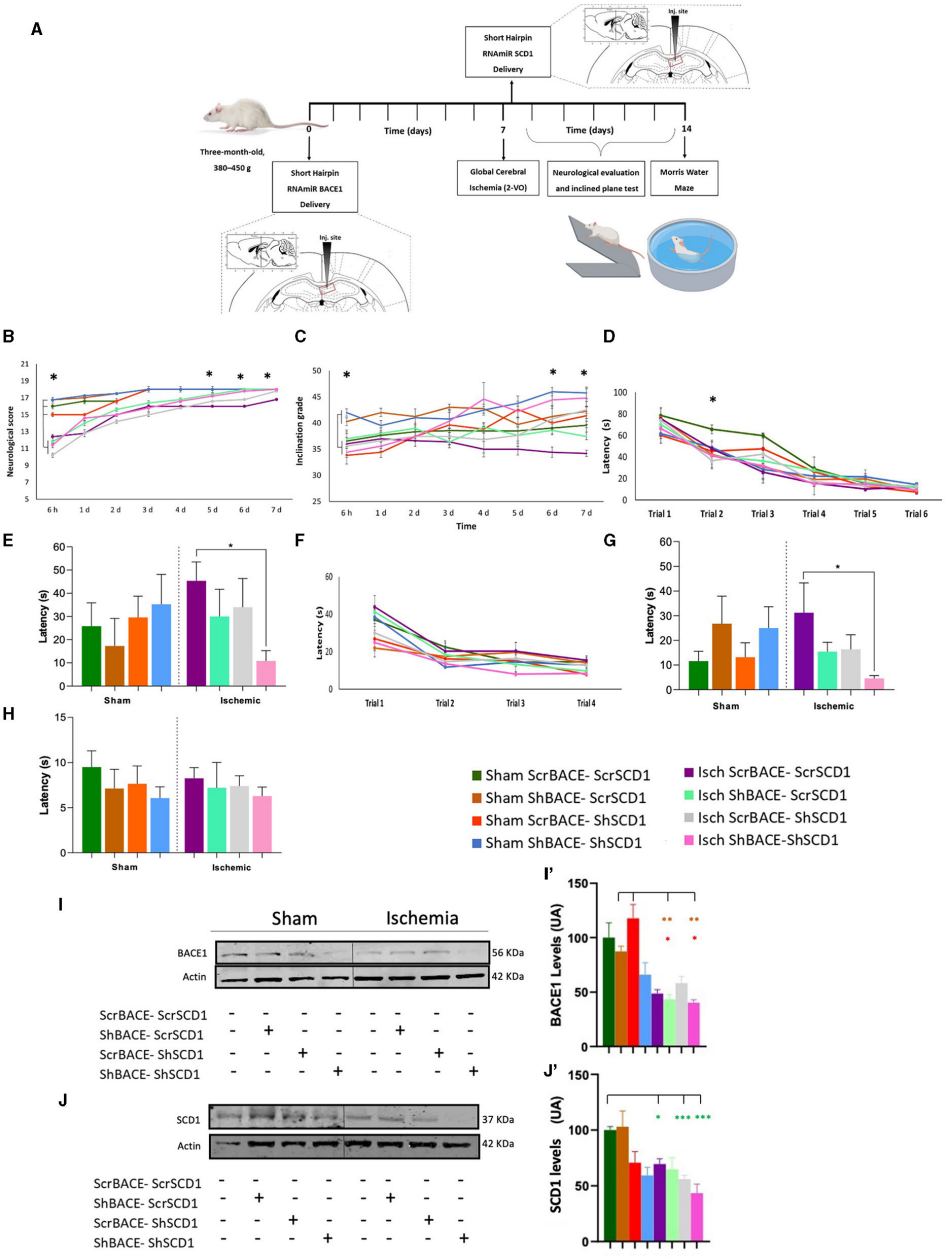
Hippocampal double silencing of BACE1 and SCD1 recovers neurological and cognitive performance in a global cerebral ischemia model in rats. **(A)** Experimental design. Scrambled, shBACE1 pre-ischemia, and shSCD1 30 min post-ischemia were injected in the hippocampus of rats. A behavioral test was realized. **(B)** Neurological score, **(C)** inclined platform for 7 days post-ischemia; **(D)** learning test was evaluated for 6 days (6 trials); and after 48 h, the **(E)** memory test. Afterward, **(F)** relearning test (4 trials, trial/day) (second position of the platform), later **(G)** transference, and **(H)** visual test. **(I)** Representative images from BACE1 and **(J)** SCD1 protein levels by Western blotting from sham and ischemic rats at the cerebral cortex under the effect of ScrBACE1-scrSCD1, shBACE1-scrSCD1, scrBACE1-shSCD1, or shBACE1-shSCD1. Arbitrary values were relativized to ß-actin expression. Brown-Forsythe and Welch ANOVA tests for comparison between groups. UA, arbitrary units. **(I')** BACE1 and **(J')** SCD1 quantification data are expressed as the group mean ± SEM. ^*^, significant difference: ^*^P < 0.05, ^**^P < 0.01, *n* = 5 animals/group. Colors in asterisks indicate significance with respect to a specific group.

We proceed to evaluate the effect of the silencing of those proteins in the hippocampus of a neurodegenerative model by ischemia in rats. Before the *in vivo* study, an *in vitro* experiment was performed where we reproduced the nuclear condensation by glutamate (purple bar), which was not avoided by the individual silencing of BACE1 (light green bar) or SCD1 (gray bar). However, the combined ShBACE1miR and shSCD1 treatment (pink bar) rescued the nuclear condensation by glutamate (purple bar) (^*^p < 0.05) (Supplementary Figure 1A) and qualitatively improved the healthy state of primary neurons in culture (Supplementary Figure 1B). Then, we determined the functional relationship between BACE1 and SCD1 by silencing both genes at the right hippocampal area in a rat model of global cerebral ischemia (Figure 2A). Global ischemia (2VO) produces tauopathy and cognitive impairment (Gutiérrez-Vargas et al., 2015; Villamil-Ortiz et al., 2016). The overall post-stroke neurological function was assessed by their performance on a scale of 18 points, where the lowest value in the test indicated the most severe injury. Neurological alterations were observed in all the ischemic groups in the first 6 h post-injury (purple, light green, gray, and pink lines) compared to sham animals (dark green, brown, blue, and orange lines) (^*^p < 0.05). Although we also observed that between the sham groups at 6 h postischemia, the reduction of BACE1 (brown) or both BACE1 and SCD1 (blue) presented a significantly better performance compared to the sham control (dark green) (^*^p < 0.05), even much better than the depletion of SCD1 alone (orange) (^**^p < 0.01). Ischemic rats injected with shBACE1-shSCD1 (pink line) exhibited a significant reduction in neurological damage, represented by a higher test score compared to sham groups, and the difference was statistically significant at days 5, 6, and 7 post-ischemia with respect to the ischemia group (purple) and Isch-ShSCD1 (gray) (^*^p < 0.05) (Figure 2B).

Interestingly, the findings obtained in the inclined platform test supported the neurological ones, where animals from sham groups with silenced BACE1 (brown) or BACE1-SCD1 (blue) started with a better score than the sham control animals (dark green) (^*^p < 0.05) and those sham with silenced SCD1 (orange) (^*^p < 0.05), which were alike to the ischemia groups (purple, light green, gray, and pink) at 6 h post-injury (Figure 2C). Nevertheless, shBACE1-shSCD1 animals showed improved motor function as assessed by the test of the inclined platform, where ischemic rats presented strong deficits to climb after 6–7 days post-ischemia (34.4° and 34.2° inclination, respectively) (purple line) compared to the sham group (scrBACE1-scrSCD1) (36.5° and 36.7° inclination) (dark green line). Interestingly, injured animals with reduced BACE1 or SCD1 expression showed milder improvements (light green line and gray line), suggesting a synergistic effect between both genes. Injection of shBACE1-shSCD1 also improved the performance of the sham group (pink line) (Figure 2C).

However, in the learning test, the experimental groups did not display differences (Figure 2D). After 2 weeks of shBACE1 injection and 1 week after ischemia and shSCD1 injection, the dual silencing of shBACE1-shSCD1 (pink bar) resulted in better spatial memory skills when compared to untreated ischemic animals (purple bar) 48 h after the last learning trial (Figure 2E, ^*^p < 0.05). Conversely, single BACE1 or SCD1 silencing (light green and gray bars) only resulted in a slight tendency to reduce the latency in the groups, showing a similar performance to the sham groups (dark green bar) (Figure 2E). During the re-learning test, no differences were found in the latency between the different groups (Figure 2F). Interestingly, the potential synergistic effect of shBACE1-shSCD1 treatment was also evidenced in the transference memory tests (pink bar), where treated animals performed significantly better than control animals (dark green bar) (Figure 2G). The visible test did not reveal any visual, motor, or motivational deficits in any experimental group (Figure 2H).

A two-way ANOVA was performed to calculate the effect of the different treatments on retention of learning, revealing that there was no statistically significant interaction between groups. However, when we realized two-way ANOVA to analyze the effect between the ischemia and each type of treatment on memory retrieval (Figure 2G), it revealed that there was not a statistically significant interaction between ischemia and shSCD1 (F (1.34) = 1.467, *p* = 0.234), but there was a statistically significant interaction between Ischemia and shBACE1 treatment (F (1.34) = 7.248, *p* = 0.011) and between Ischemia and shBACE1shSCD1 treatment (F (1.15) = 6.740, *p* = 0.02), meaning that the effect on memory retrieval was mainly by the shBACE1 treatment and supported by the shSCD1 to recover cognitive dysfunction generated by the ischemia.

On those animals analyzed for their behavioral performance, we confirmed a slight reduction of BACE1 and SCD1 by ShBACE1 and ShSCD1, respectively, and a stronger reduction of BACE1 and SCD1 by the double silencing, mainly in the ischemic cerebral cortex, from the same animals that recovered neurological and motor skills (Figures 2I, I', J, J') and whose hippocampus were processed for the next lipidome analyses.

### Reduced expression of BACE1 and SCD1 induces significant changes in lipid species containing arachidonic acid in the hippocampus, CSF, and serum from cognitively recovered rats

To gain insight into the association between BACE1-SCD1 and lipid metabolism, we performed lipidomic analysis in the hippocampus isolated from our treated and untreated ischemic rats. The initial comparison of the levels of the lipid classes analyzed did not reveal any significant differences between all groups (Supplementary Figure 2A). PCA analysis showed weak discrimination between groups. However, between the ischemic and dual treatment groups, the most discriminant species was PC 32:0 (Rho: 1.92) (Supplementary Figures 3A, B).

**Figure 3:**
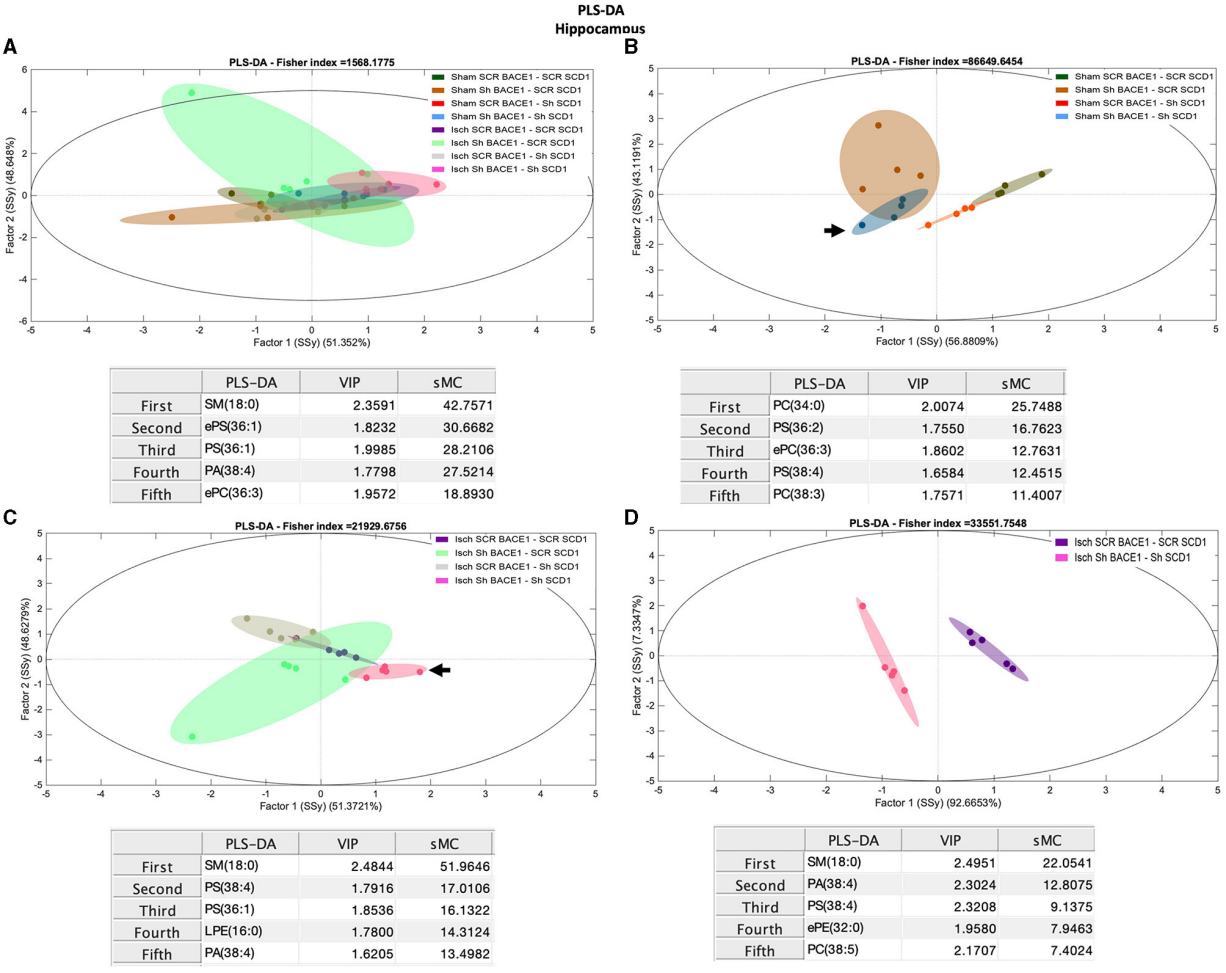
Discrimination of the lipid profile of the hippocampus from cognitive improvement in an animal model silenced for BACE1 and SCD1 genes. Multivariate analyses of the lipid profiles from the **A–D** hippocampus. Score plots of PLS-DA and partial least squares analysis to discriminate between the lipid classes also show the projections of the data with the sMC and VIP metrics and values from sMC and VIP in a table per item. The variables in the analyses are as follows: Sham ScrBACE1-scrSCD1, Sham shBACE1-scrSCD1, Sham scrBACE1-shSCD1, Sham shBACE1-shSCD1; Isch ScrBACE1-scrSCD1, Isch shBACE1-scrSCD1, Isch scrBACE1-shSCD1, Isch shBACE1-shSCD1. PA, phosphatidic acid; PC, phosphatidylcholine; LPC, lysophosphatidylcholine; ePC, ether phosphatidylcholine; PS, phosphatidylserine; ePS, ether phosphatidylserine; PE, phosphatidylethanolamine; LPE, lysophosphatidylethanolamine; ePE, ether phosphatidylethanolamine; PI, phosphatidylinositol; PG, phosphatidylglycerol; SM, sphingomyelin, *n* = 5 per group.

PLS-DA also showed limited capacity to discriminate between the different groups (Figure 3A). However, the dual treatment Isch-shBACE1-shSCD1 exhibited the greatest separation from the others (arrow), based on specific species with higher scores by the sMC index [sphingomyelin (SM) 18:0 (42.75), monounsaturated phosphatidylserine (PS), and ether PS (ePS) 36:1 (sMC: 30.67 and 28.20, respectively), and arachidonate associated with phosphatidic acid (PA 38:4, sMC: 27.52)] (Figure 3A). Likewise, PLS-DA analysis was also able to classify untreated sham and ischemic groups from those treated with the double shBACE1-shSCD1 silencers (sham or Isch srcBACE1-scrSCD1) (Figures 3B, C; arrows).

Our analysis also showed that, in the ischemic group, separation between untreated and shBACE1-shSCD1-treated clusters was mostly prompted by differences in SM 18:0 (sMC: 51.9), PS 38:4 (sMC: 17), PA 38:4 (sMC: 13.5), and saturated and monounsaturated PLs, such as PS 36:1 (sMC: 16.13) and LPE 16:0 (14.31) (Figure 3C).

To validate these results, we compared Isch-srcBACE1-scrSCD1 and Isch-shBACE1-shSCD1 using PLS-DA analysis. The main discriminant lipid molecular species between these two groups were the saturated lipid species SM 18:0 (sMC: 22.05) and ePE 32:0 (16:0/16:0) (sMC: 7.9) and PLs associated with AA (PA 38:4, PS 38:4) (sMC: 12.8 and 9.13, respectively) (Figure 3D). In support of these data, sham or ischemic groups treated with single shBACE1 or shSCD1 silencers could also be discriminated from their untreated counterparts based on different species containing AA and other very long fatty acids (Figures 3B, C). In contrast, when shBACE1 and shSCD1 were silenced under sham or ischemic conditions, segregation between groups was achieved by changes in PLs containing palmitic (16:0) and stearic (18:0) acids (Figure 3D).

**Figure 4:**
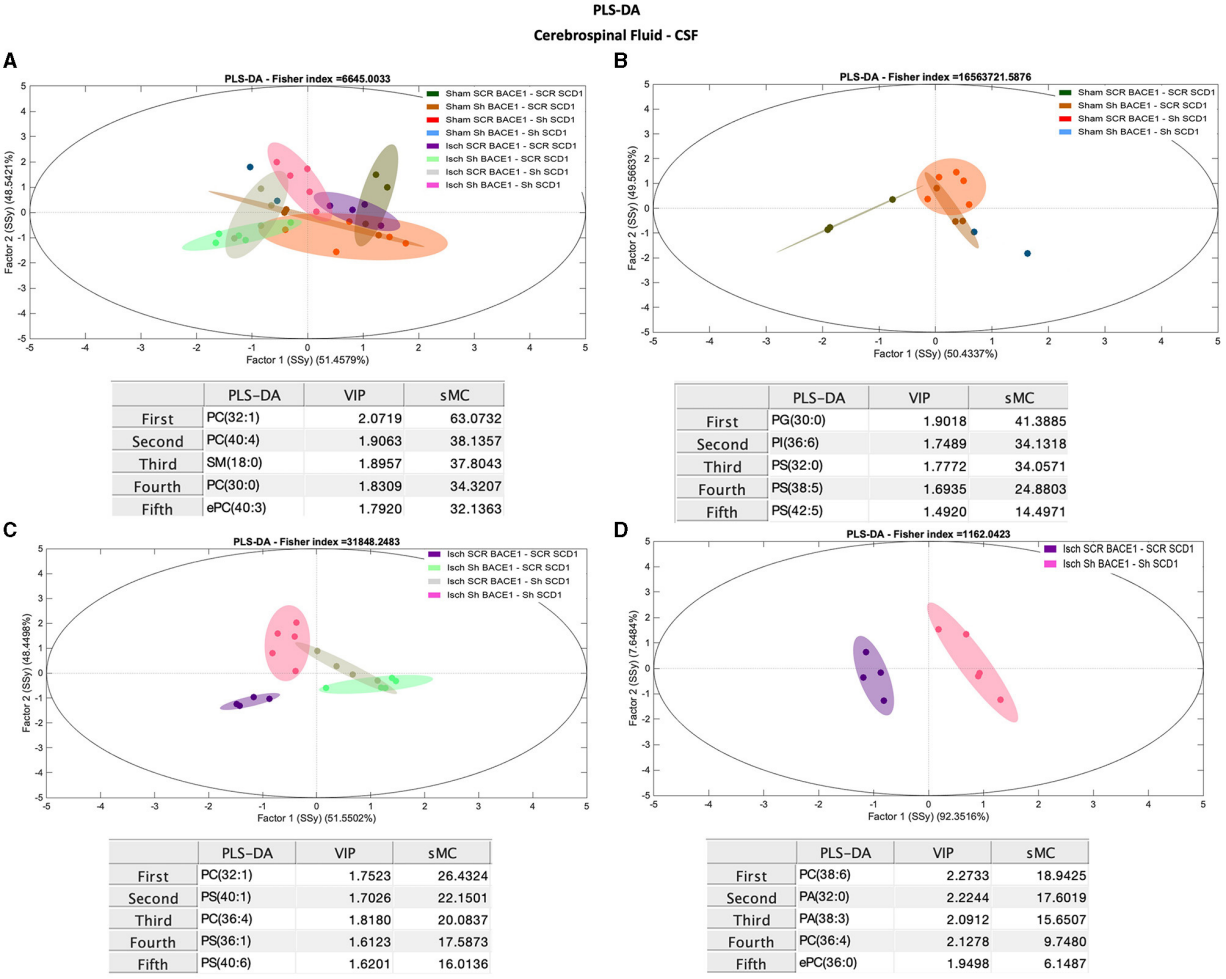
Discrimination of the lipid profile of the CSF from cognitive improvement in animal models silenced for BACE1 and SCD1 genes. Multivariate analyses of the lipid profiles from the **A–D** CSF. Score plots of PLS-DA and partial least squares analysis to discriminate between the lipid classes also show the projections of the data with the sMC and VIP metrics and values from sMC and VIP in a table per item. The variables in the analyses are as follows: Sham ScrBACE1-scrSCD1, Sham shBACE1-scrSCD1, Sham scrBACE1-shSCD1, Sham shBACE1-shSCD1; Isch ScrBACE1-scrSCD1, Isch shBACE1-scrSCD1, Isch scrBACE1-shSCD1, Isch shBACE1-shSCD1. PA, phosphatidic acid; PC, phosphatidylcholine; LPC, lysophosphatidylcholine; ePC, ether phosphatidylcholine; PS, phosphatidylserine; ePS, ether phosphatidylserine; PE, phosphatidylethanolamine; LPE, lysophosphatidylethanolamine; ePE, ether phosphatidylethanolamine; PI, phosphatidylinositol; PG, phosphatidylglycerol; SM, sphingomyelin, *n* = 5 per group.

Conversely, comparisons between groups for different lipid classes in the CSF did not show robust differences in the % molar concentration (Supplementary Figure 2B). But PCA analysis showed a general discrimination of PC 32:0 (Rho: 26.58) and PC 34:0 (Rho: 10.35) between the ischemic and dual treatment groups (Supplementary Figure 3D), among other species of PC. However, PLS-DA results using lipidomic data from CSF samples successfully classified Isch scrambled and Isch double-silenced groups, mainly influenced by changes in saturated and monounsaturated PLs and lipid species containing AA (Figures 4A–D). Specifically, the main discriminant PL species between both groups were PC 38:6 (18:2/20:4) (sMC: 18.9), PA 38:3 (18:0/20:3) (sMC: 15.7), PC 36:4 (16:0/20:4) (sMC: 9.7), and PA 32:0 (16:0/16:0) (sMC: 16.6) (Figure 4D).

Interestingly, changes in monounsaturated PLs (PC 32:1), PS (40:1, PS 36:1), and PLs associated with polyunsaturated fatty acids (PUFAs) (PC 36:4) and PS (40:6) also contributed to discriminate between CSF from untreated ischemic animals vs. those treated with shBACE1 or shSCD1 independently (Figure 4C).

### Depletion of BACE1 and SCD1 regulated the composition and concentration of PC/LPC in the serum of cognitively recovered rats

**Figure 5:**
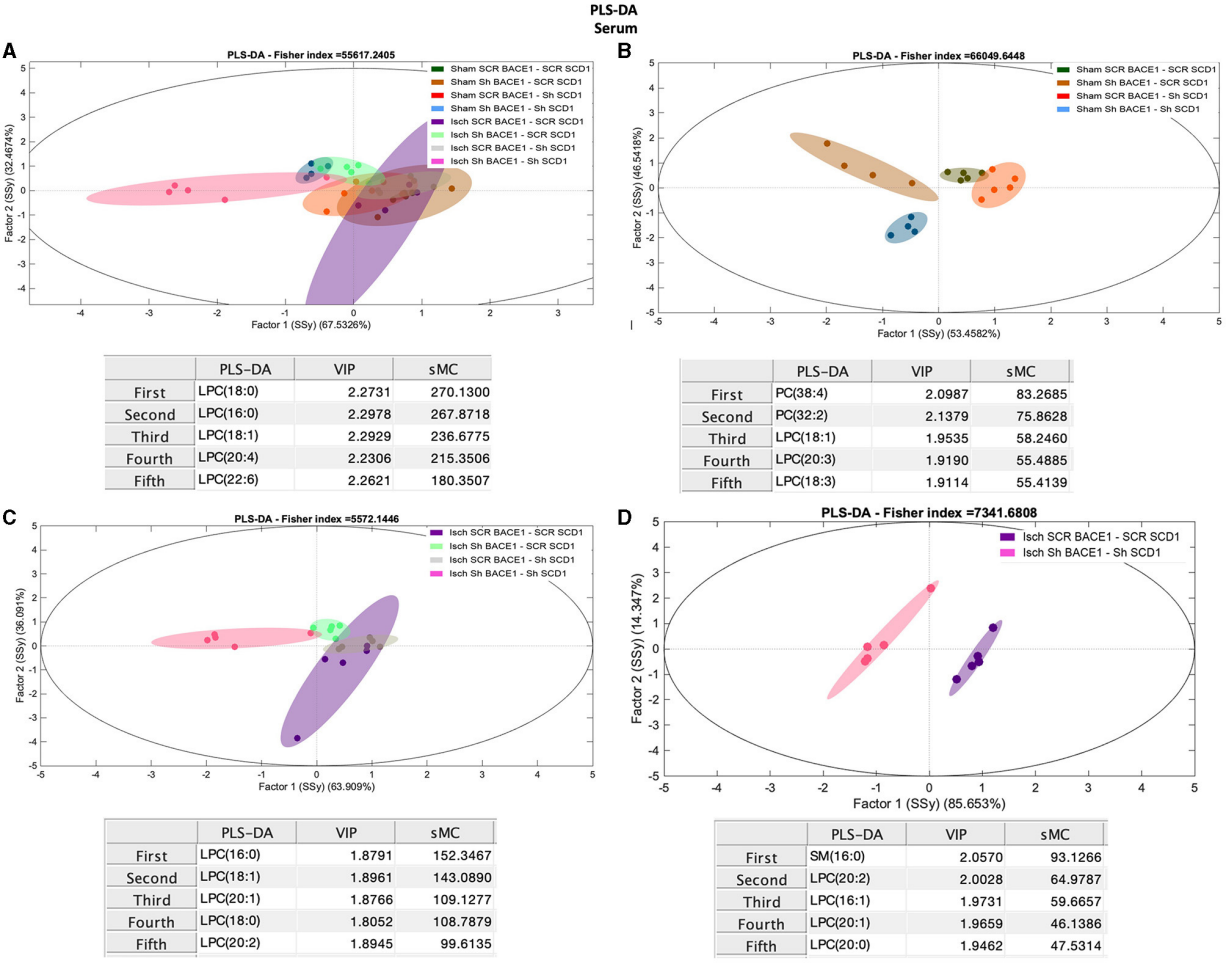
Discrimination of the lipid profile of the hippocampus, CSF, and serum from cognitive improvement in an animal model silenced for BACE1 and SCD1 genes. Multivariate analyses of the lipid profiles from the **A–D** serum. Score plots of PLS-DA partial least squares analysis to discriminate between the lipid classes also show the projections of the data with the sMC and VIP metrics and values from sMC and VIP in a table per item. The variables in the analyses are as follows: Sham ScrBACE1-scrSCD1, Sham shBACE1-scrSCD1, Sham scrBACE1-shSCD1, Sham shBACE1-shSCD1; Isch ScrBACE1-scrSCD1, Isch shBACE1-scrSCD1, Isch scrBACE1-shSCD1, Isch shBACE1-shSCD1. PA, phosphatidic acid; PC, phosphatidylcholine; LPC, lysophosphatidylcholine; ePC, ether phosphatidylcholine; PS, phosphatidylserine; ePS, ether phosphatidylserine; PE, phosphatidylethanolamine; LPE, lysophosphatidylethanolamine; ePE, ether phosphatidylethanolamine; PI, phosphatidylinositol; PG, phosphatidylglycerol; SM, sphingomyelin, *n* = 5 per group.

**Figure 6:**
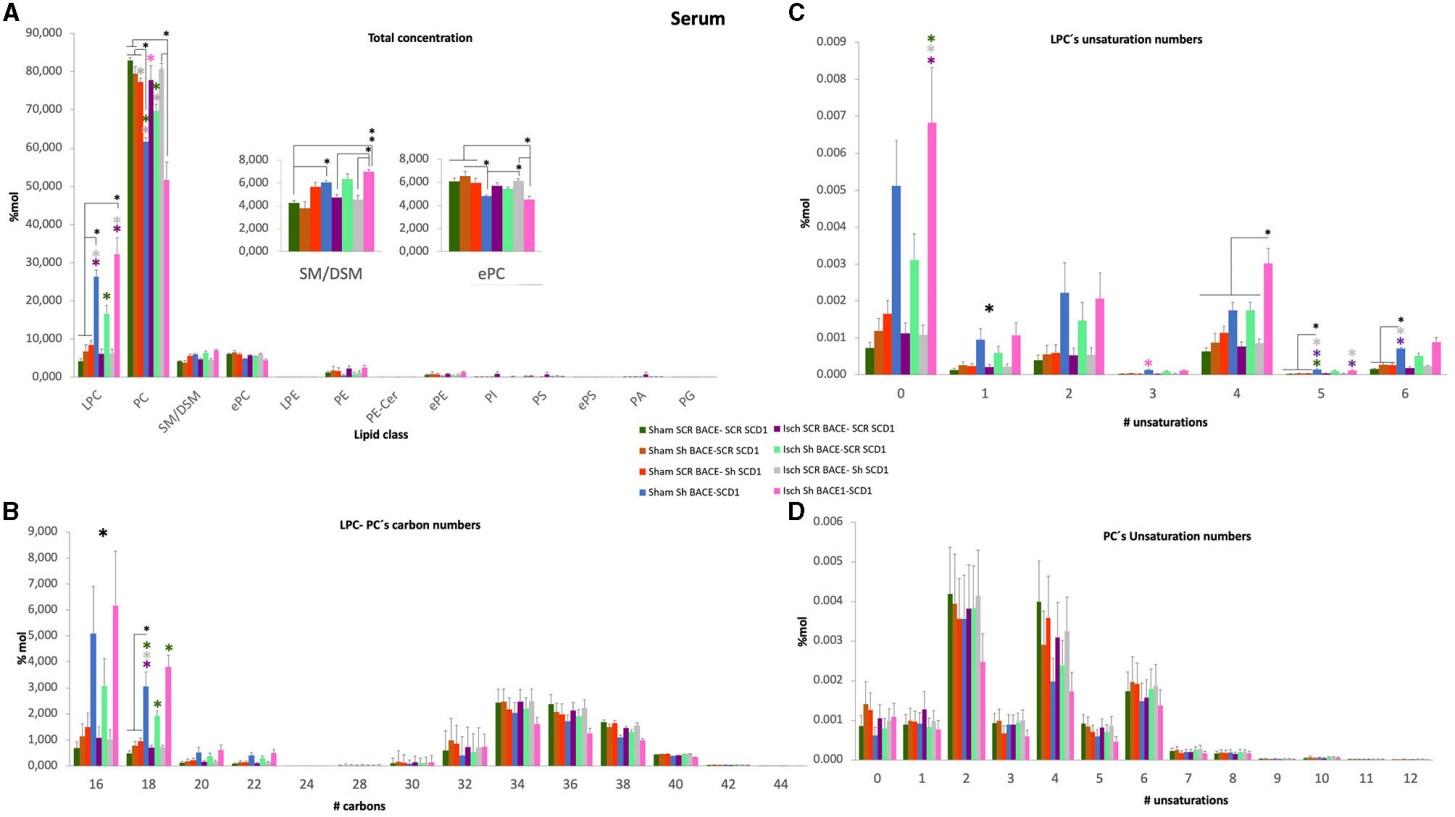
General changes of total concentration, carbon length, and unsaturations of PLs from the serum of cognitively recovered animal models silenced for BACE1 and SCD1 genes. **(A)** The lipid class profiles are expressed as % Mol composition observed in the serum. **(B)** LPC-PC chain lengths equal to the total number of carbon atoms in the fatty acid moieties; **(C)** LPC and **(D)** PC unsaturations equal to the total number of double bonds in the fatty acid moieties for the analyzed lipid extract from serum. All lipid species were measured (means), and the error bars represent the SEM. ANOVA or Kruskall-Wallis test followed by Tukey's or Dunnett's T3 *post-hoc* test according to the normality test. ^*^p < 0.05, ^**^p < 0.01, ^***^p < 0.001; a single large black asterisk means that there are differences between groups, but not by *post-hoc* test. Color asterisks indicate differences between compared groups, according to the color bar. The data are expressed as % Mol. *n* = 5 per group. The variables in the analyses are as follows: sham ScrBACE1-scrSCD1, sham shBACE1-scrSCD1, sham scrBACE1-shSCD1, sham shBACE1-shSCD1; Isch ScrBACE1-scrSCD1, Isch shBACE1-scrSCD1, Isch scrBACE1-shSCD1, Isch shBACE1-shSCD1.

Finally, we compared the lipid composition of serum samples from treated and untreated sham and ischemic animals. Contrary to the hippocampus and CSF, the analysis of serum samples showed significant changes in the concentration of LPC and PC between all groups (Figure 5A), where shBACE-shSCD1 treatment generated an inverse effect, increasing 3- to 6-fold the % Mol of LPC and decreasing 1.6-fold the % Mol of PC compared with the rest of the groups. In particular, Isch shBACE1-shSCD1 and Isch scrambled groups were mainly classified according to changes in LPC species containing saturated and monounsaturated palmitic and stearic acid, such as LPC 18:0 (sMC 270.1), LPC 16:0 (sMC 267.9), LPC 18:1 (sMC 236.7), and PUFAs such as LPC 20:4 (sMC 215.3) and LPC 22:6 (sMC 180.3) (Figures 5A, D). These results were supported by the effects of shBACE1 and shSCD1 in the sham and ischemic groups, highlighting the association between PLs as PC and LPC, and FA saturation to changes under shBACE1-shSCD1 expression was exacerbated in the serum (Figures 5B, C; Figure 6A; with respect to Supplementary Figure 2). Interestingly, the PCA showed similar effects in the double-silenced ischemic group, discriminating similar PLs and composition in serum than the PLS-DA approach, such as LPC 16:0 (Rho 60.52), LPC 18:0 (Rho 46.33), and PC 36:4 (34.44) (Supplementary Figure 3F).

Next, we decided to use the lipidomic data to determine the effect of treatments on the number of carbons and unsaturation of all PL classes in the hippocampus, CSF, and serum samples; however, we did not find significant changes in the hippocampus and CSF. However, when we focused on serum, the previous changes detected in the PC and LPC classes were revealed. Although we did find significant changes in the number of carbons and the number of double-bonds in total PC (Figures 6B–D), we detected that the silencing of BACE1 alone on ischemia or dual BACE1-SCD1 gene silencing in sham or ischemic groups significantly increased the concentration of carbons and mainly the number of unsaturations in the LPC class (Figures 6B, C). In this sense, shBACE1 shSCD1 treatment on ischemic rats (pink bar) compared to the untreated Isch-Scr group (purple bar) increased FA of 16 carbons from 1 to 6% Mol (6X); FA of 18 carbons from 0.8 to 4 % Mol (5X) (Figure 6B) and FA with 0 unsaturation from 0.0012 to 0.0068 % Mol (5.7X) (Figure 6C).

**Figure 7:**
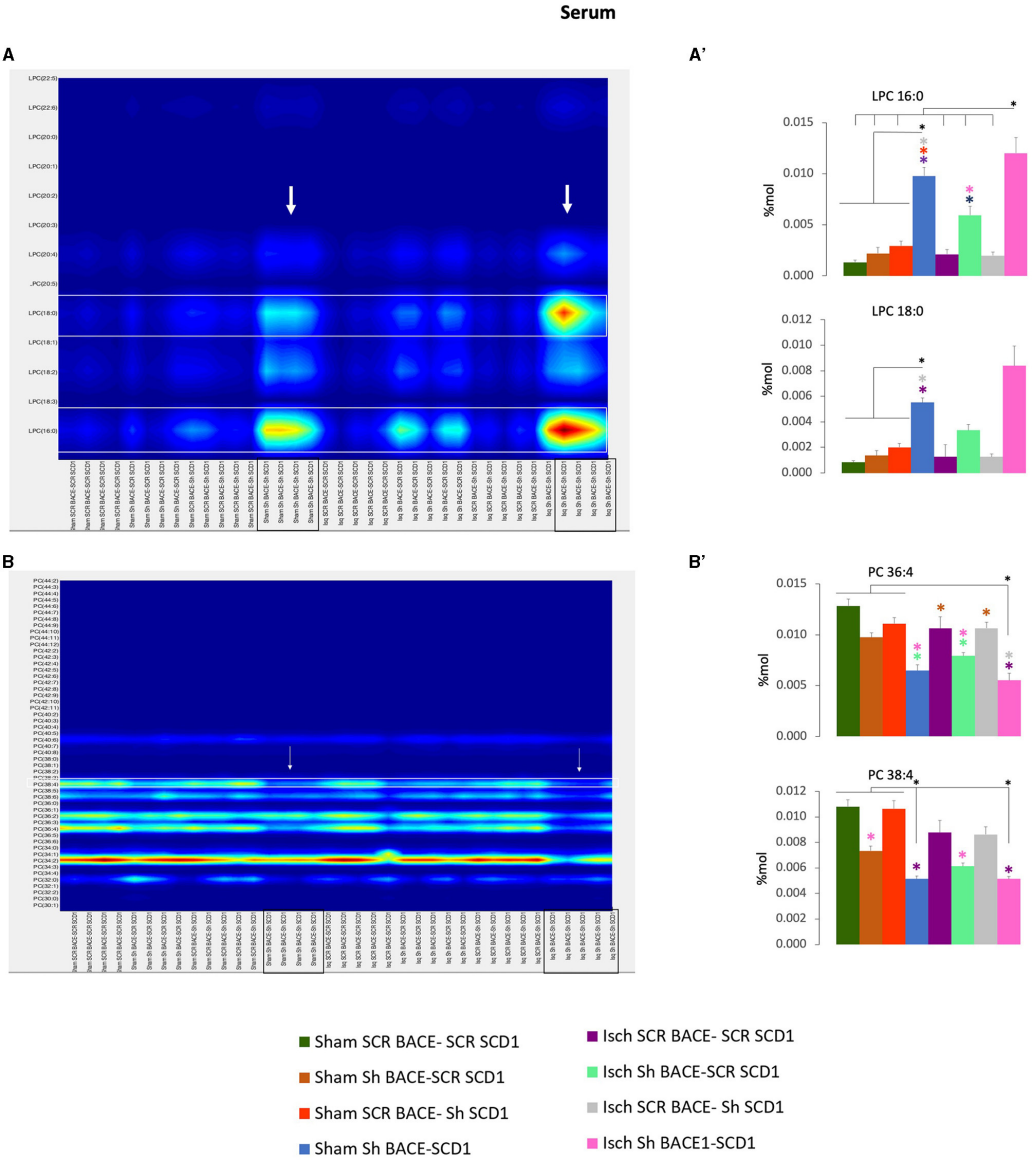
Fatty acid composition and abundance of LPC and PC were modified by the double silencing of BACE1 and SCD1 in cognitively recovered rats. The chain lengths are equal to the total number of carbon atoms in the fatty acid moieties, and the saturations are equal to the total number of double bonds in the fatty acid moieties of PC and PC from serum. Contour plots of the more influential subclasses of **(A)** LPC and **(B)** PC are shown. The respective % Mol changes by the different experimental groups affect the **(A')** LPC and **(B')** PC compositions. ANOVA or Kruskall-Wallis test followed by Tukey's or Dunnett's T3 *post-hoc* test according to the normality test. The data were expressed as % Mol. *n* = 5 per group. Color asterisks mean: ^*^p < 0.05, between compared groups, according to the color bar. The variables in the analyses were as follows: sham ScrBACE1-scrSCD1, sham shBACE1-scrSCD1, sham scrBACE1-shSCD1, sham shBACE1-shSCD1; Isch ScrBACE1-scrSCD1, Isch shBACE1-scrSCD1, Isch scrBACE1- shSCD1, Isch shBACE1-shSCD1.

In particular, the shBACE1shSCD1 treatment significantly modified the increase of LPC 16:0 (0.002 to 0.012 % Mol) (6X) and LPC 18:0 (0.0018 to 0.0085 % Mol) (4.7X) with respect to ischemic scrambled rats (Figures 7A, A') and inversely, in those same groups, the shBACE1shSCD1 treatment reduced PC 38:4 (0.0085 to 0.0045 % Mol) (2X) and PC 36:4 (0.011 to 0.005 % Mol) (2.2X) (Figures 7B, B'), both of which supported species with the heavier sMC rescued by PLSDA on serum when all groups were compared (Figure 5A).

### Depletion of BACE1 and SCD1 has differential effects on LPC/PC levels in neural and endothelial cells associated with protection

When we analyze the effect of BACE1 and SCD1 depletion in astrocytes on coculture with neurons or endothelial cells. In general, we found an increase of GFAP immunoreactivity (IR) by glutamate (Supplementary Figures 4B, B', E), without changes in LDH release or condensed nuclei in astrocytes, but there was a tendency or significant reduction of GFAP-IR, mainly by the double inhibition of BACE1 and SCD1. Interestingly, the neurons also affected by glutamate presented a significant reduction of the condensed nuclei when those were in coculture with depleted astrocytes for SCD1 or for SCD1/BACE1 (Supplementary Figure 5C), accompanied by a redistribution of SCD1 IF in neurites either in controls or under glutamate toxicity (Supplementary Figures 5A, A', B, B').

Complementarily, astrocytes in coculture with endothelial cells showed a significant increase of GFAP and SCD1 IF by glutamate (Supplementary Figures 6A, B, E, F) with a significant increase of LDH release without changes in the condensed nuclei (Supplementary Figures 6C, D), which was reversed by the double depletion of SCD1 and BACE1 (Supplementary Figures 6B, C, E, F). In the same experiment, the endothelial cells in coculture with astrocytes, showed a disruption of cell membrane integrity labeled by ZO-1 IF and a significant increase in SCD1 IF at the soma by the glutamate treatment (Supplementary Figures 7, B, B', D). Although the coculture with astrocytes depleted for SCD1 or BACE1/SCD1 prevented the loss of membrane cell integrity in spite of the glutamate exposure (Supplementary Figures 7B, D).

Surprisingly, lipid extract from those same cells in cocultures showed a differential regulation of phospholipid levels focused on an inverse relationship between PC and its plasmalogen LPC in a cell type-dependent manner (Supplementary Figures 8A–C), which was highlighted by the ratio of LPC/PC under the double depletion of BACE1 and SCD1 in the context of cell protection against glutamate toxicity. In concordance with the morphological findings shown in the cocultures of astrocytes-neurons (Supplementary Figures 4, 5) and astrocytes-endothelial cells (Supplementary Figures 6, 7), LPC/PC ratio levels increased in astrocytes and endothelial cells and reduced in neurons in protected conditions. Also, the inhibition of SCD1 induced an increase of LPC/PC in endothelial cells (Supplementary Figures 8D–F).

### Accumulation of PC in AD *in vitro* models

**Figure 8:**
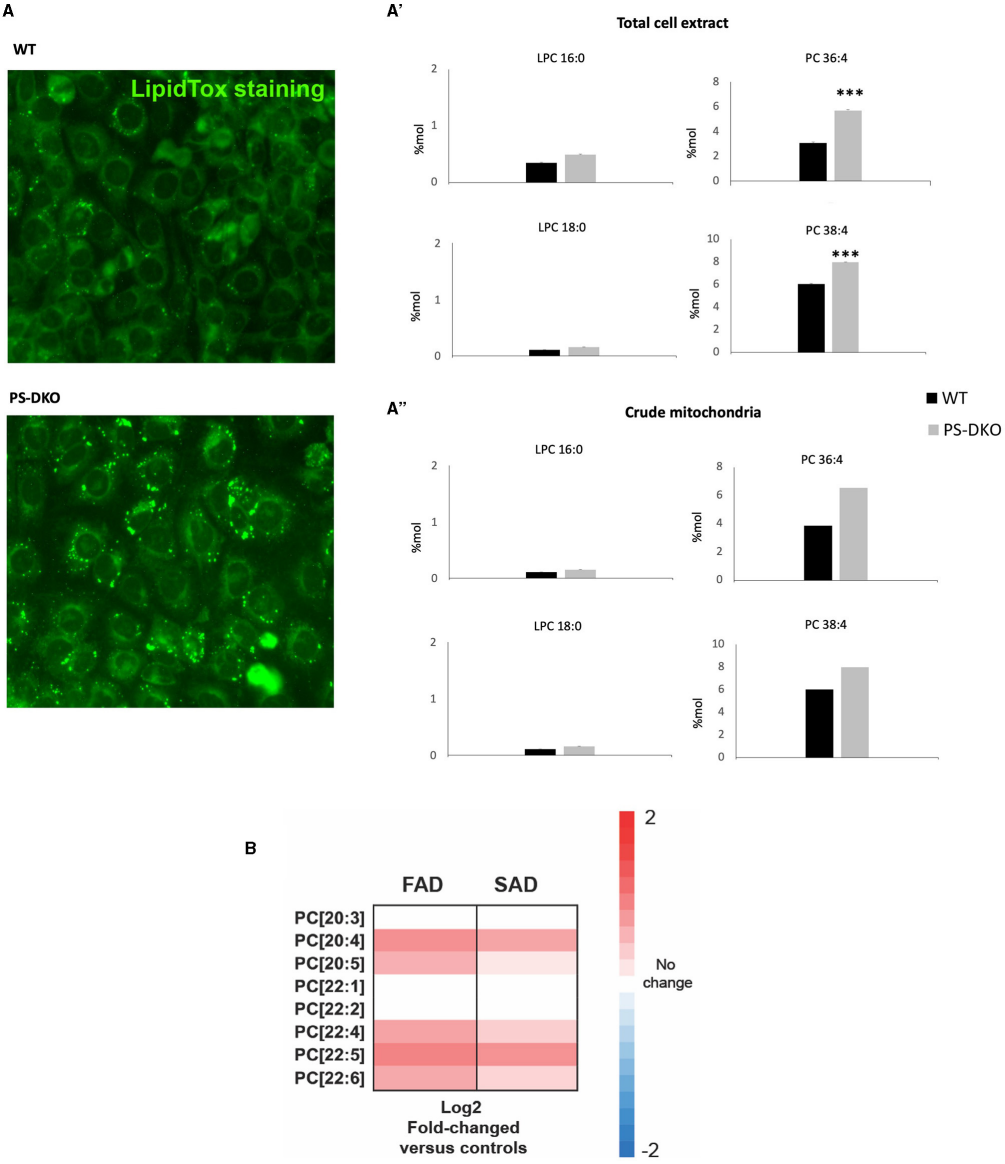
PC-PUFAs changes verified in AD mice models and human AD samples. **(A)** Lipid droplets representative staining from WT and PS-DKO mice's fibroblasts, **(A')** LPC and PC % Mol from total cell extract, and **(A”)** LPC and PC % Mol from crude mitochondria of WT and PS-DKO mice's fibroblasts. **(B)** Analysis of cells from FAD and SAD patients (*n* = 20 biological replicates/group). The data were expressed as % Mol. ^***^p < 0.0001. Hot map: Blue = −2, White = 0, Red = 2.

Interestingly, overregulated levels of PC 36:4 and PC 38:4 were associated with lipotoxicity in a fibroblast culture from Presenilin double knockout mice (PS-DKO). Specifically, we found a significant increase in the levels of PC 36:4 and PC 38:4 without changes in LPC in total homogenates from PS-DKO fibroblasts (Figure 8A') and a lesser extent in mitochondrial fractions (Figure 8A”) under the context of lipotoxicity (Figure 8A). Furthermore, analysis of cells from FAD and SAD patients recapitulated those changes in the concentration of specific PC species, such as PC 20:4, and associated with other PUFAs, as in PS-DKO cells (Figure 8B).

### Histological analysis of hippocampal tissues from AD and CADASIL patients shows significant alterations in the levels of BACE1 and SCD1

**Figure 9:**
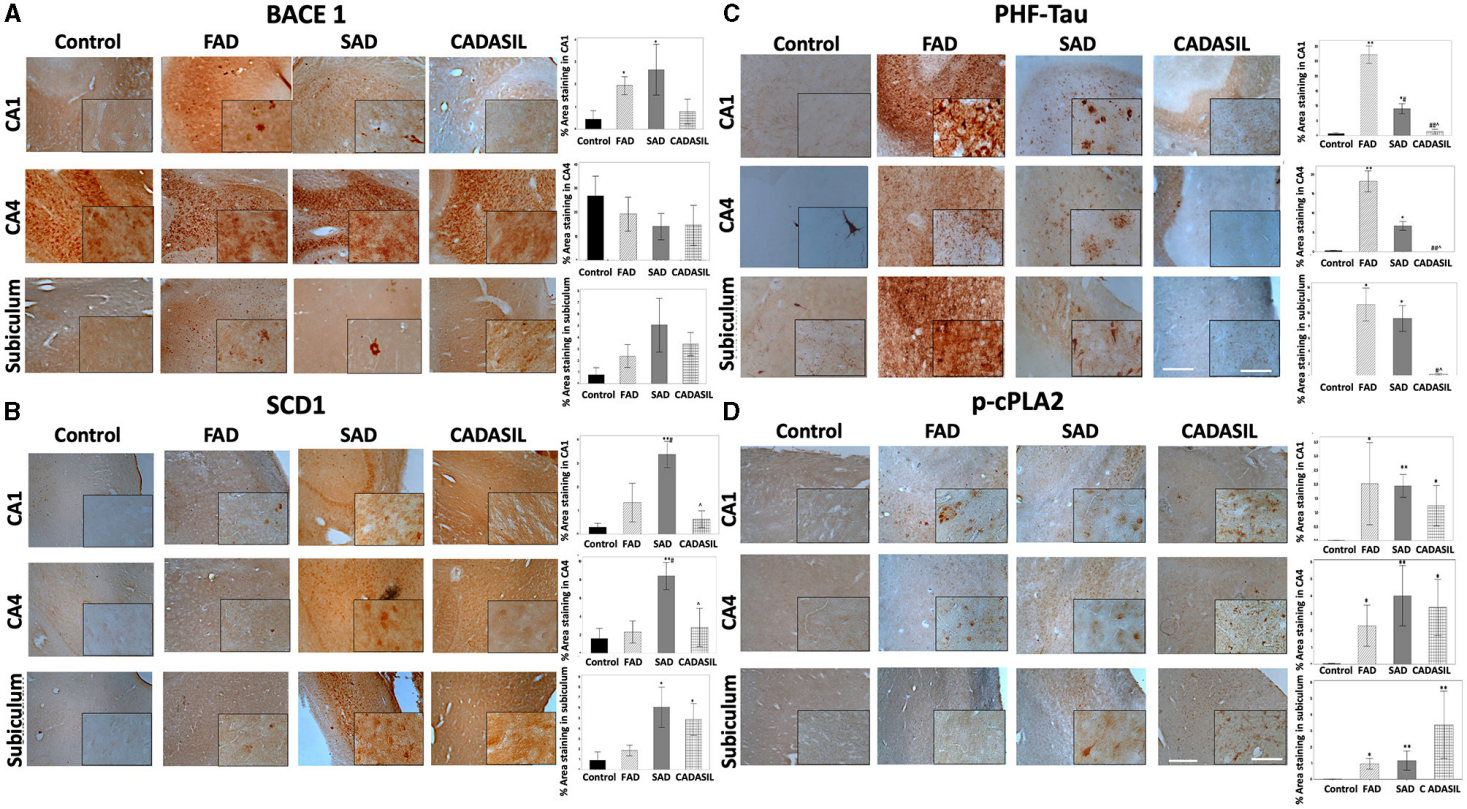
BACE1, SCD1, PHF-tau, and cPLA2 immunohistochemistry staining in the hippocampus of human dementia brains. **(A)** % stained area for BACE1, **(B)** SCD1, **(C)** PHF-tau, and **(D)** *p*-cPLA2; for control, FAD, SAD, and CADASIL groups in CA1, CA4, and subiculum area. Representative images 10×, bar graph: 100μm; insets: 40 ×, bar graph: 50μm. The bars indicate the SEM values. Kruskal-Wallis test was used for independent samples. ^*^p ≤ 0.05, ^**^p ≤ 0.001 compared to control; #*p* ≤ 0.05 compared to FAD; ^∧^*p* ≤ 0.05 compared to SAD; ##p ≤ 0.001, #p ≤ 0.05 compared to FAD; ^∧^*p* ≤ 0.05 compared to SAD. The number of cases: control (*n* = 5), FAD (*n* = 5), SAD (*n* = 10), and CADASIL group (*n* = 5).

The expression of BACE1 is increased in AD; however, specific regional changes in levels of BACE1 have not yet been confirmed. We performed an immunohistochemical analysis to reveal BACE1 in the hippocampus (CA1 and CA4 areas) and subiculum from FAD, SAD, and CADASIL patients (the most common form of familial brain arteriopathy) and healthy controls. We observed a significant increase in BACE1 levels in CA1 from FAD (^*^p = 0.007) and SAD (^*^p = 0.006) compared to controls, as well as in FAD (^*^p = 0.020) and SAD tissues (^*^p = 0.021) compared to those from the CADASIL group (Figure 9A). However, no differences were found between CADASIL (*p* = 0.720) and controls or between FAD (*p* = 0.704) and SAD at CA1 (Figure 9A). We did not find any significant differences in BACE1 expression in the CA4 (*p* = 0.854) (Figure 9A) or subiculum areas (*p* = 0.065) between the different groups (Figure 9A). Notably, BACE1 showed a higher and more homogeneous expression in CA4 than in CA1 and subiculum regions for all experimental groups. In addition, BACE1 was expressed in all brain areas under analysis in FAD, SAD, and CADASIL tissues, while healthy tissues showed particularly higher levels of this protein in CA4 regions (Figure 9A), as shown previously by others (Laird et al., 2005; Xue et al., 2015). Using the same tissue samples as before, we determined the expression and distribu tion of SCD1 by immunostaining. We observed a significant increase in SCD1 levels in CA1 and CA4 areas of SAD (^**^p = 0.0001) patients compared to FAD cases (#*p* = 0.044) and controls (Figure 1B). Moreover, we did not find any significant differences between FAD (*p* = 0.197) and controls or CADASIL (*p* = 0.636) and controls in the same CA1 and CA4 areas. In contrast, SAD (^*^p = 0.006) and CADASIL (^*^p = 0.023) showed higher SCD1 expression in the subiculum areas compared to controls or FADs (Figure 9B). No significant differences were found between the FAD samples (*p* = 0.465) and the controls (Figure 9B). Notably, healthy tissues showed overall low SCD1 expression, and the SAD and CADASIL tissues presented with markedly higher SCD1 levels in different brain areas.

Our immunohistochemical study also revealed significant changes in the expression of p-cPLA2 between all types of dementia in the study and the control groups. Specifically, CA1, CA4, and the subiculum areas showed significantly higher expression in FAD [CA1 (p^*^= 0.032), CA4 (p^*^= 0.048), and subiculum (p^*^= 0.025)], SAD [CA1 (p^**^= 0.001), CA4 (p^**^= 0.001), subiculum (p^**^= 0.006)], and CADASIL [CA1 (p^*^ = 0.041), CA4 (p^*^ = 0.016), subiculum (p^**^ = 0.003)] (Figure 9D).

As a control, we also assessed the degree of tau phosphorylation in our samples by staining with the anti-phospho tau antibody AT8 (which recognizes phosphorylation in serine 202, threonine 205, and serine 208 as the main phosphorylated positions involved in the PHF). As expected, we observed increased PHF-tau labeling in CA1 and CA4 areas of FAD [CA1 (^**^p = 0.000; CA4 (^**^p = 0.001)] and SAD patients [CA1 (^*^p = 0.027), CA4 (^*^p = 0.041)] compared to controls (Figure 9C) or CADASIL samples (^**^p = 0.001; ^*^p = 0.031, respectively). The latter showed no significant differences when compared to the control group (*p* = 0.966) in either CA1 or CA4. Subiculum areas showed essentially the same PHF-tau staining pattern as CA1 and CA4 regions (Figure 9C) in FAD (^*^p = 0.003), SAD (^*^p = 0.005), and CADASIL (*p* = 0.698) compared to controls. As observed in Figure 9C, PHF-tau expression was higher in FAD than in SAD, and this difference was more marked in the CA1 (#*p* = 0.044) and subiculum than in CA4.

### BACE1 and SCD1 colocalize in brain tissues from SAD patients

**Figure 10:**
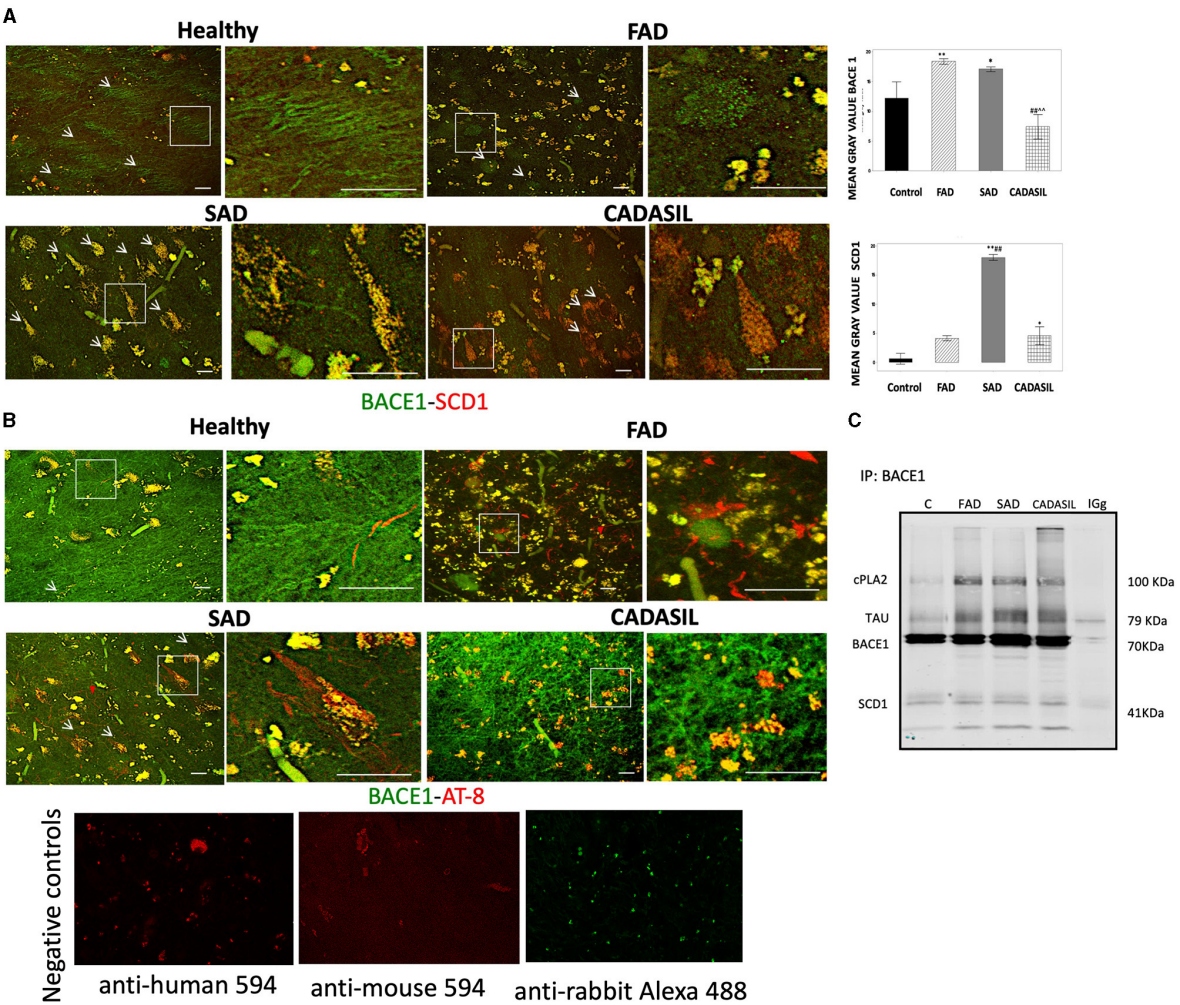
BACE1, SCD1, and PHF-tau immunofluorescences in CA1 of dementia brains. **(A)** Representative images from the maximal projection of BACE1 (green) and SCD1 (red). The merge and colocalization filter (yellow) for each condition is shown. The magnification of every white box is shown to the right of each image. The mean gray value of every positive signal for BACE1 and SCD1 is plotted in the bar diagrams (top right), as is the quantification of colocalization. **(B)** BACE1 (green) and PHF-tau (AT-8) (red) from CA1 immunostaining. Bar graph: 50μm. Arbitrary units of fluorescence intensity. Error bars indicate SEM of means. Negative controls staining without primary antibodies are shown in **(B)**. Kruskal-Wallis's test for independent samples was used. ^*^p < 0.05; ^**^p < 0.001, compared to control; ##*p* < 0.001 compared to FAD; ^∧^∧*p* < 0.001 compared to SAD. **(C)** Molecular complex by immunoprecipitation of BACE1 and blotting detection of cPLA2, PHF, and SCD1. Number of cases: control, *n* = 5; FAD, *n* = 5; SAD, *n* = 10; CADASIL, *n* = 5. Number of total positive signals for BACE1: control, *n* = 25; FAD, *n* = 955; SAD, *n* = 1,749; CADASIL, *n* = 52. Number of total positive signals of SCD1: control, *n* = 45; FAD, *n* = 734; SAD, *n* = 1,809; CADASIL, *n* = 23.

Our immunostaining results indicate that CA1 areas showed the most significant changes in the expression of BACE and SCD1 between all cases and control groups. Therefore, we decided to analyze the distribution of these two proteins by double IF staining in this area of the hippocampus. Each positive fluorescent signal for BACE1 or SCD1 was manually selected as an ROI in the maximal projection image. The number and mean gray value for each ROI in the green (BACE1) and red (SCD1) channels were obtained using the ImageJ software (NIH) (Figure 10A). The mean gray value for each protein was expressed as the difference between the mean gray value obtained for each ROI and the mean gray value for the background of the corresponding channel.

Our results showed a significant increase in the mean gray value for each positive BACE1 signal for the FAD (^**^p = 0.001) and SAD (^*^p = 0.052) cases compared to controls (Figure 10A) and with respect to the CADASIL samples [FAD (##*p* = 0.000), SAD (^∧^∧*p* = 0.001)]. No significant differences were observed between FAD and SAD cases (*p* = 0.052) or between CADASIL (*p* = 0.261) and controls (Figure 10A).

On the other hand, the mean gray value for each SCD1 positive signal was greater in SAD (^**^p = 0.000) and CADASIL (^*^p = 0.025) than in the controls and FAD cases. Furthermore, we found a significant association (*p* = 0.0001) when both mean gray values for BACE1 and SCD1 were analyzed in each ROI (Figure 10A). Our data show a particular increase in colocalization between BACE1 (green channel, white arrows) and SCD1 (red channel, red arrows) in FAD and SAD cases (Figure 10A).

### BACE1/SCD1 are associated with the PHF-tau/cPLA2+ molecular complex in the CA1 area of dementia brains

We performed a double IF for BACE1 and PHF-tau markers (Figure 10B) to confirm that both proteins are in the same cells, as previously described in AD brains by Duyckaerts et al. (2008). Our data showed that both markers were in close apposition in FAD tissues (Figure 10B) and partially overlapped in SAD and CADASIL samples (insets, Figure 10B). Interestingly, BACE1 IP suggests a potential molecular complex or at least a coaggregation between SCD1, PHF-tau, cPLA2, and BACE1, which are markedly amplified in dementia cases compared to the control samples (Figure 10C).

## Discussion

In this study, we found for the first time the association between BACE1 and SCD1 in neurodegeneration. We found that BACE1 and SCD1 are increased in neurodegenerative conditions *in vivo, in vitro*, and in human brains. BACE1 and SCD1 associations had a close relationship with cPLA2 and PHF-tau in the human brain with dementias (FAD, SAD, and CADASIL), mainly in FAD and SAD. *In vivo* BACE1 and SCD1 augments were associated with astrogliosis in the hippocampus of ischemic rats. The BACE1/SCD1 relationship was supported by a significant effect of the dual silencing of BACE1 and SCD1 genes producing a synergy on motor and cognitive recovery in a global cerebral ischemia rat model, impacting the phospholipid profile from the hippocampus, CSF, and mainly serum. In addition, this double silencing in the sham and ischemic groups induced a proportional inverse ratio between total PC and LPC in serum, represented mainly by the reduction of PC 38:4 and PC 36:4 and the increase in LPC 16:0 and LPC 18:0 in the neurologically recovered rats. Our results were also backed up by a differential effect of BACE1/SCD1 depletion in a neural and endothelial cell type mode-dependent regulation of LPC/PC ratio, which was inverse to the neurotoxic *in vitro* or pathological *in vivo* conditions. Dysregulation of PC in a neurodegenerative context was supported by an increase in PC 36:4 and 38:4 in AD models, such as in PS1-DKO cells under lipotoxicity and in cells from FAD and SAD cases. Together, these data suggest a novel convergence between BACE1 and SCD1 in the pathogenesis of neurodegeneration related to pro-inflammatory phospholipids.

Previous studies have shown that SFAs are a risk factor for developing AD (Morris et al., 2003) and that MUFAs are increased in the brains of individuals with AD (Astarita et al., 2011). Furthermore, the fatty acid (FA) regulation of BACE1 has a potential effect on the upregulated activity of amyloidosis (Marwarha et al., 2019). In addition, high levels of MUFAs and stearyl-coA desaturase 1 (SCD1) have been described in the cortex and hippocampus of patients with AD (Astarita et al., 2011). In this study, we demonstrated differential SCD1 expression between SAD and FAD. This difference may suggest that greater levels of SCD1 could act as a possible causative agent of AD in individuals with SAD who do not have predisposing mutations associated with early-onset AD. Additionally, our results also showed significantly higher SCD1 expression in the subiculum of CADASIL compared to that in controls. Elevated lipoprotein (a) (Lp(a)) levels have been established in patients with CADASIL (Gong et al., 2010), and dyslipidemia and an altered lipid profile (Haley et al., 2020) are associated with ischemic stroke. Desaturase enzymes have been associated with chronic diseases; however, to date, there has not been a reported imbalance in the expression of SCD1 in CADASIL cases.

On the other side, cytosolic phospholipase A2 (cPLA2) is a ubiquitously distributed enzyme that cleaves membrane glycerophospholipids to form AA. AA is converted to a potent inflammatory lipid mediator; therefore, cPLA2 has been implicated in diverse cellular responses, such as inflammation and cytotoxicity (Li et al., 2019). Besides, it has been seen that its increased activity may directly cause loss of membrane integrity, excessive production of fatty acids, and oxidative stress (Liu et al., 2014). Elevated expression of p-cPLA2 in AD brains has not been previously demonstrated. Other researchers have shown that cPLA2 activation promotes neurodegeneration and that the genetic ablation of cPLA2 improves cognitive function and protects against the toxic effects of Aβ oligomers in a mouse model of FAD (Desbène et al., 2012). It has also been reported that soluble Aβ oligomers activate cPLA2 through transient relocalization of cPLA2 to the plasma membrane, which would generate the production of AA from membrane phospholipids and induce neuronal apoptosis (Malaplate-Armand et al., 2006). These results would suggest that the elevated Aß levels typical of FAD and SAD cases could raise the levels of p-cPLA2 found in these dementias. Importantly, when analyzing the levels of p-cPLA2 in brains with CADASIL, we found that they were significantly higher in all the areas studied compared to the controls. Although this finding is novel in CADASIL brains, previous studies have associated cPLA2 activity with ischemia. This is because cPLA2 activity results in the production of proinflammatory lipid mediators. In fact, it has been shown that cPLA2α is a crucial component in the pathway of stroke injury (Bonventre, 1996) and that the inhibition of c-PLA2 attenuates focal ischemic brain damage in mice with cerebral ischemia-reperfusion injury (Bonventre, 1996; Liu et al., 2017).

On the other hand, either SCD1 or BACE1 is synthesized in the ER and subsequently undergoes traffic through the endosome system. The IP results in this study suggest that SCD1 and BACE1 may be close or interact in both compartments. In addition, normal SCD1 activity is necessary for the formation of autophagosomes (Ishigami et al., 2018). Autophagosomes fuse with endosomes and lysosomes sequentially to form autolysosomes. In this context of the cellular membrane-trafficking system, abnormal levels of SCD1 could be altering the membranes of this vesicle system necessary for the transport and normal function of BACE1. For their part, the phospholipases A2 (PLA2) are key enzymes of phospholipid degradation and crucial in maintaining the membrane composition. BACE1 is ultimately degraded within lysosomes, which are surrounded by a phospholipid membrane, vulnerable to the activation of PLAs (Li et al., 2019). Moreover, recent studies have reported that cPLA2 activation leads to lysosomal damage, causing neuronal autophagosome accumulation and neuronal death (Li et al., 2019). In addition, it could also cause an impediment to the degradation of BACE1 and a higher production of Aß. In addition, since Aß activates cPLA2 (Malaplate-Armand et al., 2006), this could create a vicious cycle.

Another interesting perspective is that cPLA2 activation joints endosomes and ER (Yang et al., 2018), which would favor the convergence between both BACE1 and SCD1 in a lipid environment as lipid rafts, mainly on mitochondria-associated ER membranes (MAMs) (Weng et al., 2006; Agrawal et al., 2020; L. Zheng L. et al., 2021). In addition to cholesterol and sphingomyelin, this location also contains either the C99 or SCD1 product of BACE1 cleavage (Kawarabayashi et al., 2004), as well as ACSL4, which is an active enzyme related to phospholipid composition (Smith et al., 2013) by PUFA incorporation into PLs (Kuwata and Hara, 2019). Furthermore, previous studies have suggested a convergence between cPLA2 and PHF-tau (Sundaram et al., 2012) and BACE1 and PHF (Villamil-Ortiz et al., 2018). However, if BACE1 has a potential causative relationship with SCD1 and phospholipid regulation, triggering proinflammation and neurodegeneration has not been approached before.

In this last sense, it is widely known that BACE1 and PHF are upregulated in dementia and are highly associated upstream with the proinflammatory cascade surrounding the Aß hypothesis. However, deposition of Aß or its elimination is not completely correlated with the prevention of dementia or the recovery of brain function affected by AD (Lozupone et al., 2020). Therefore, the search for alternative explanations has mainly focused on the events involved in the trigger of SAD and also given a stem or broader role to BACE1. Our previous studies have shown that the silencing of BACE1 increases Hsc70/LAMP2 in lipid rafts, producing a reduction of PHF with the consequent cognitive improvement of 3xTg AD mice (Ravaut et al., 2020). Moreover, this effect was indirectly related to the maturation of autophagosomes by lipidation of phosphatidylethanolamine (PE), which was blocked by 3-methyadenine (3-MA), preventing the action of BACE1 targeting PHF-tau elimination (Ravaut et al., 2020). In addition, silencing of BACE1 achieved the regulation of plasmalogens such as LPE and ePE, which were recovered from the AD environment in the hippocampus of a 3xTgAD mouse model, mainly its FA composition modifying 18:0 for 18:1 and 20:4 for 22:6, in the context of proinflammatory reversion reducing cPLA2 and COX-2 (Villamil-Ortiz et al., 2016).

Additionally, BACE1 targeting reverses PHF and proinflammation in neurons in an SCD1-dependent manner (Villamil-Ortiz et al., 2018). All these previous studies are in concordance with our current data, confirming the interaction of BACE1 and SCD1 in human dementia brains. In addition, the current findings confirm the upregulation of BACE1 and SCD1 associated with astrogliosis by ischemia in the rat hippocampus. Likewise, BACE1 is also expressed in reactive astrocytes associated with disrupted vessels, mainly in SAD and closer to PHF-tau+ cells, which could be a potential tissue scene in the parenchymal pathogenesis (Chacón-Quintero et al., 2021). However, further cause-and-effect experiments should be conducted in the future to further investigate this interaction and its physiological consequences.

Nevertheless, the double silencing of BACE1 and SCD1 induced neurological and cognitive improvement in ischemic rats. The shBACE1 treatment was the main factor responsible for memory retrieval improvement in ischemic rats, supported by the shSCD1 treatment. Those findings were supported by the lipid profile discrimination, which was generated mainly by shBACE1 alone in ischemic rats and accentuated by shSCD1 (Figures 3B, C, 4C, 5B, C).

Complementarily, PC 32:0 in the hippocampus and PC 34:0 in the CSF had the higher Rho index, 1.92 and 26.57, respectively, by PCA (Supplementary Figures 3B, D). Where both lipid classes have been reported as protectors of the inflammatory response (J. Zheng J. et al., 2021), inversely to the adverse effect of LPC 16:0 (Huo et al., 2020; Zheng L. et al., 2021), which in our study were detected in the circulation. This correlation was also supported by our previous observations of a proinflammatory environment in the brain and serum in an ischemia rat model (Sabogal-Guáqueta et al., 2018).

In addition, a heavier discrimination of PLs was associated with SM 18:0, and PLs were associated with arachidonic derivatives [PA (38:4), PS (38:4, 36:4)] by PLS-DA. The cleavage products of SM, SCD1, and BACE1, such as C-99, are present in lipid rafts, mainly on MAM, whose overexpression correlates with AD pathogenesis (Agrawal et al., 2020; Wang et al., 2021). Moreover, cPLA2 activation is located closer to the ER-mitochondria-overproducing MAMs in AD, which activates another enzyme that produces the incorporation of PUFAs on PLs, such as ACSL4 (Acyl-CoA synthetase long-chain family member 4), and an increase in arachidonic derivatives (Kuwata and Hara, 2019). ACSL4 has a preference for ARA, forming ARA-CoA, which is incorporated into lysophospholipids by lysophospholipid acyl transferases and has also been associated with cognitive impairment (Lee et al., 2012) and, in a few cases, neuroprotection (Sambra et al., 2021). Moreover, our previous studies showed that PS 40:7 and plasmalogens such as LPE and LPC were associated with AA in the human cerebral cortex from dementias (SAD and CADASIL) with respect to healthy brains, in addition to the discrimination of PC 42:7 and PC 44:11 in CSF from patients with dementia (Sabogal-Guáqueta et al., 2020). Furthermore, the reduction of PC and the increase of LPC in serum have been associated with neuroprotection in humans (Angelini et al., 2014), as well as the accretion of PC associated with PUFAs to proinflammation (Kim et al., 2014), which was backed up by the increase of PC 36:4 and 38: in AD *in vitro* models in addition to their reduction by shBACE1 and shSCD1 in the sham and ischemia contexts in our study.

BACE1 and SCD1 depletion could prevent the increase of cPLA2, an enzyme participating in the land cycle brain-blood (J. Zheng J. et al., 2021), avoiding the accumulation of pro-inflammatory lipid classes in the brain, and clarifying adverse lipid species in circulation, which is because we detect a smaller increase of LPC 22:6 by shBACE1shSCD1 at the ischemic hippocampus, supported by a reduction of LPC/PC in protected neurons from glutamate in coculture with double-depleted astrocytes, although inversely to neurons, both astrocytes and endothelial cells presented an increase in it under protection, which is also correlated with an increase in saturated LPC in the serum of recovered rats. It has been described that LPC-DHA, through the land cycle, is transported by albumin from the blood to the brain through the BBB mainly by the MSFD2 receptor, improving brain function (Nguyen et al., 2014), and especially PUFAs are associated with a decrease in the risk of dementia (Rapoport et al., 2001; Smith and Nagura, 2001; Sambra et al., 2021), which complementarily might support the neurological recovery obtained in our model.

Considering that the knockdown of BACE1 and SCD1 imply a reduction and not a complete depletion of the gene expressions. Also, our data showed the presence of other species in serum, such as 18:1, 18:2, and 18:3. Which could be related to the most abundant LPCs in human plasma, that in addition to LPC palmitate (16:0), LPC stearate (18:0); those are LPC oleate (18:1), LPC linoleate (18:2) and LPC eicosatetraenoate (20:4), and other species included LPC eicosapentaenoate (20:5), and LPC docosahexaenoate (22:6; DHA) (Croset et al., 2000). The sources of LPCs in human plasma include LPCs directly absorbed from the diet. LPC is generated by phospholipase A2 (PLA2) activity on PC in membranes during digestion and then absorbed in the gut (Lands' cycle) (Wang and Tontonoz, 2019). Complementarily, the reduction of palmitoleic (16:1) and oleic (18.1) induces reacylation (ALJohani et al., 2017; Xu et al., 2022), increasing the action of phospholipases, activating the land cycle, and supplying the LPC-PUFAs. This study focuses future attention on BACE1 and SCD1 in LPC metabolism and the land cycle.

However, the accumulated PUFAs are associated with peroxisome deficiency (Poulos et al., 1988) and demyelination (Abe et al., 2014), which would suggest an improvement of ß-oxidation in the peroxisomes on the parenchyma and a consequent discrimination of arachidonic derivatives in CSF and serum by the double silencing of BACE1/SCD1. In concordance with preliminary data, we detected that silencing of BACE1 recovered the hydroxyacyl-CoA dehydrogenase trifunctional multienzyme complex subunit alpha (HADHA), an enzyme that is incharge of the long-chain PUFA ß-oxidation and was affected by glutamate in neuronal cultures (Villamil-Ortiz et al., unpublished). In addition, targeting BACE1 increased the activation of mTOR (Piedrahita et al., 2016). This pathway has been related to lipid metabolism sensing (Wang et al., 2021) and autophagy (Chrienova et al., 2021). Therefore, our data could suggest the BACE1/SCD1 depletion implies degradation of VLPUFAS and less incorporation of PUFAs in the intent of homeostasis recovery, but more detailed studies should be conducted for a better understanding.

At the end, there was an evident cooperation and potentiation by the silencing of both genes, BACE1 and SCD1, on the increase of LPC C16-22: 0–6 from serum, achieving a discrimination cloud from all groups represented by a larger projection of LPC 18:0, 16:0 with the higher score (sMC: 270, 267.86, respectively) and by the counter plot, stronger than the effect, reducing PC 38:4 and PC 36:4, whose alteration in PC 36:4 and PC 38:4 was reproduced in PS-DKO cells, and surprisingly those were recapitulate in human FAD and SAD cells, mainly represented by an increase of PC 20:4, PC 20:5, and PC 22:6.

Together, our data could be explained because the silencing of BACE1 and SCD1 may reduce C99, SM, cholesterol, and SCD1 in lipid rafts (mainly MAM) (Piedrahita et al., 2016; Montesinos et al., 2020), which could trigger a reduction of ACSL 4, 5, and 6, preventing the incorporation of PUFAs into PLs and improving the ß-oxidation of VLPUFAS, which promotes the release of FAs of C16, C18:0 to the serum, favoring the inverse levels of LPC/PC. However, additional and deeper studies must be conducted to understand the role of the BACE1/SCD1 complex in the regulation of peripheral vs. central metabolism.

In summary, BACE1 and SCD1 associations had a close relationship in neurodegeneration in *in vitro* and *in vivo* models and in human dementia brains. When BACE1 and SCD1 were silenced, there was a discrimination of phospholipids associated with arachidonic acid and other PUFAs in the hippocampus, CSF, and serum of ischemic animals with improved neurological and cognitive skills, related to a reduction of PC 36:4 and PC 38:4. Those results were backed up by an upregulation of PC 38:4 and PC 36:4 found in an AD *in vitro* model and FAD and SAD cells. Therefore, the data suggest a novel convergence of BACE1 and SCD1 in neurodegeneration, related to pro-inflammatory phospholipids.

## Data availability statement

The raw data supporting the conclusions of this article will be made available by the authors, without undue reservation.

## Ethics statement

The studies involving humans were approved by BioEthical Committee for Human Studies from the University of Antioquia. The studies were conducted in accordance with the local legislation and institutional requirements. The participants provided their written informed consent to participate in this study. The animal study was approved by Ethics Committee for Animal Experimentation of the University of Antioquia, Medellin, Colombia. The study was conducted in accordance with the local legislation and institutional requirements.

## Author contributions

FAB-G, AMB-S, MP-H, IDS-C, JAG-V, and GPC-G designed experiments. FAB-G, MP-H, IDS-C, AMB-S, JAG-V, JDA-L, EA-G, and GPC-G analyzed the data and reviewed and edited the manuscript. MP-H and JV-O realized human tissue experiments. AMB-S and JAG-V performed in vivo and in vitro experiments, respectively. CAV-L contributed to the sampling and post-mortem diagnosis of human tissue. EA-G did PS-DKO in vitro experiments. EA-G contributed PS-DKO cells and FAD and SAD cells from human AD. FAB-G, MP-H, IDS-C, AMB-S, JAG-V, JDA-L, EA-G, and GPC-G wrote the manuscript. All authors contributed to the article and approved the submitted version.
