# Supplementary material for: BACE1 and SCD1 are associated with neurodegeneration
Source: Front Aging Neurosci. 2023 Sep 8;15:1194203. doi: 10.3389/fnagi.2023.1194203 (PMC10516302; doi:10.3389/fnagi.2023.1194203)
Supplement: Supplementary file 1 [file Data_Sheet_1.DOCX]

**Appendix I:**

**Material and methods:**

**Primary culture of astrocytes**

Astrocytes cultures were obtained from cortices of P1 neonates Wistar rats. Cortices were dissected using a 0.25% trypsin/EDTA mixture (Gibco, 15400054, Canada) for 15 min. Then these were centrifuged at 3000 rpm for 5 minutes and trypsin/EDTA solution was removed. The remaining cell material was resuspended in DMEM (Sigma-Aldrich), cultured in T75 flasks and maintained in DMEM medium supplemented with 10% FBS with a 1% antibiotic mix (Gibco). After, the cells were incubated at 37°C to 5% CO_2_. Culture medium was changed every two days. At DIV 8 to 10, the flasks were shaken at 350 rpm for three days with intermediate changes of fresh DMEM medium to remove other type of cells (microglia and oligodendrocytes). Astrocytes were subcultured to 12-well plates for co-culture use at a density of 1.5 X10^5^ cells per well.

**Endothelial cell cultures**

Endothelial cell line bEnd.3 (ATCC CRL-2299) from murine brain microvasculature was used as a model of vascular endothelium as previously described (Becerra-Calixto et al., 2018). bEnd.3 cells were thawed in 100 mm (p100) glass polystyrene (petri) dishes in DMEM (Sigma-Aldrich) supplemented with fetal bovine serum (20% FBS) and 1% penicillin antibiotic mix -streptomycin (Gibco), and brought to 37°C to 5% CO_2_. After 24 hours the medium was replaced by maintenance medium (DMEM with 10% SBF and 1% penicillin-streptomycin) and kept at 37°C to 5% CO2. Subcultures were performed using 0.25% trypsin/EDTA (Gibco, 15400054) for 5 minutes and after subcultured into 12-well dishes with gelatin polymer-coated slices and brought to a density of 1.5 × 10^5^ cells per well.

**Primary culture of neurons**

Neurons were obtained from cortices E18-19 Wistar rat embryions. These cortices were dissected using a 0.25% trypsin/EDTA mixture (Gibco, 15400054) for 15 minutes. They were then centrifuged at 3000 rpm for 5 minutes and trypsin/EDTA solution was removed. Cell material was resuspended in platting medium (DMEM, 10% horse serum, and 1% penicillin-streptomycin) and cultured in 12-well dishes with poly-L-lysine-coated crystals (Sigma-Aldrich, St, Louis, MO, USA) and paraffin dots on the border. Cell concentration was 1.5X10^5^ cells per well, and after two hours, the medium was replaced by neurobasal medium (Gibco ™ 21103049, Rockville, MD, USA), which contained B-27 supplement (Sigma-Aldrich), chicken egg albumin (Sigma-Aldrich) and a 1% antibiotic mixture of penicillin-streptomycin (Gibco) and cytosine arabinoside (AraC) at a concentration of 500 nM to prevent glial cell growth. The next day, the AraC was diluted to 50 nM and the cells were maintained under these conditions until cocultivation with the astrocytes.

**Coculture of astrocytes and endothelial cells**

Paraffin dots allowed that slices with astrocytes at DIV15 were overlapped with bEnd.3 cells previously subcultured, without direct contact and allowing to share the culture medium, kept at 37°C with 5% CO2. After 6 days the coculture was disassembled to inhibit BACE1 and SCD1 only in astrocytes and after 24 hours, the coculture was assembled and treated with 125 μM glutamate (Becerra-Calixto et al., 2018). At DIV23, the culture medium was collected to measure cytotoxicity and both types of cells were fixed for immunofluorescence staining.

**Coculture of astrocytes and neurons**

Astrocytes at DIV15 and neurons at DIV7 were cocultured in a sandwich slice supported in paraffin dots, without contact cells to cells and maintained in neurobasal medium at 37°C with 5% CO2. After 6 days the coculture was disassembled to inhibit BACE1 and SCD1 only in astrocytes and after 24 hours, the coculture was assembled and treated with 125 μM glutamate (Becerra-Calixto et al., 2018). 48 hours later the culture medium was collected to measure cytotoxicity and both types of cells were fixed for immunofluorescence staining.

***In vitro* treatments and measurements:**

Astrocytes were treated at DIV21 with inhibitor IV Beta-secretase (CAS 797035-11-1) at 1 μM and with the SCD1 inhibitor (CAY 10566) at 10 μM. Twenty-four hours later the cocultures (astrocytes-neurons and astrocytes-endothelium) were treated with 125 μM glutamate for 20 minutes.

*Cytotoxicity* was measured by release of lactate dehydrogenase (LDH) using LDH Cytotoxicity Detection Kit (Roche Diagnostics GmbH), in agreement to instructions. After incubation samples were measured at a wavelength of 490 nm in a microplate reader (BIO RAD iMARKTM). The absorbance value was used to calculate the percentage of LDH release with the following formule: % LDH Release= ((A-low control)/(high control-low control)) * 100, where A was the value of the absorbance indicating the level of LDH activity for each sample, the low control was the absorbance value indicating the basal activity of LDH released by untreated cells, and the high control was the maximum measure of LDH release by the cells treated with 1% Triton X-100.

For *in vitro* *immunofluorescence*, cell cultures were fixed with 4% paraformaldehyde in cytoskeleton buffer with sucrose (CBS) (Posada-Duque et al., 2017). Autofluorescence was removed using 50 mM ammonium chloride (NH4Cl) for 10 minutes. Cells were then permeabilized with 0.2% Triton X-100 prepared in CBS and subsequently treated with 2.5% FBS in CBS to block nonspecific antigen-antibody binding. Cultures were incubated overnight at 4°C with 1:500 mouse monoclonal antibody against SCD1 (E-8 Santa Cruz Biotechnology) for astrocytes, neurons and endothelium, 1:250 rabbit polyclonal antibody against GFAP (Invitrogen) for astrocytes. And 1:400 rat monoclonal antibody against zonula occludens (ZO-1) (Santa Cruz Biotechnology) in bEnd.3 cells. The next day, cultures were incubated for 30 minutes in the absence of light with Alexa 488 or Alexa 594 secondary antibodies (Molecular Probes, 1:500), the cytoskeleton was stained with Alexa 594-conjugated phalloidin probe (1:500, Molecular Probes) for neurons and the nucleus was stained with Hoechst 33258 (Invitrogen, 1:5000) for all three cell types. Finally, three serial washes with phosphate buffered saline (PBS) were performed and the crystals with the immunolabeled cells were fixed to slides with FluorSave. Cells were observed with an Olympus IX 81 epifluorescence microscope and 20 images were captured with immersion oil in the 60X, NA 1.42 objective for each of the treatment groups. Each image was made up of three channels: blue, red and green, plus a “merge” made up of the previous ones. Finally, the images were quantified using the ImageJ (NIH) software.

***In vitro* morphological analysis**

The average number of *condensed nuclei* was quantified using the “Count and measure objects” tool of the Image-Pro Plus software, where nuclei with a diameter between 3.0 and 6.0 μm were defined as condensed. The percentage of condensed nuclei was calculated by formule: % condensed nuclei = [(condensed nuclei/ (condensed nuclei + normal nuclei)] ×100.

For *Quantification of fluorescence intensity*, the background of each image was subtracted from an arbitrary sector that did not contain cells using the “subtract” tool of the ImageJ (NIH) software. Then, the fluorescence intensity (IF) was measured for SCD1 in neurons, astrocytes and endothelium, and for GFAP in astrocytes, according to the intensity value using the “Measure tool” in the ImageJ software (NIH) for the respective fluorescence channel. Values were relativized over the area of the cell, and pseudocolor was assigned per channel.

*Fluorograms* were used to determine the distribution of SCD1 at the three cell types. A 50 μm line was drawn through the cells, crossing the membrane, cytoplasm, and nucleus with the help of the “Line profile” tool of the ImageJ (NIH) software. This procedure was performed on 20 cells per assay (n=4) and finally the most representative profile for each treatment was chosen.

**Figure legends:**

**Supplementary Figure 1. BACE1 and SCD1 silencing did not affect neurons under glutamate toxicity**. Primary cortex neurons were doubly transduced with SCR, BACE1 shRNA-miR and SCD1shRNA and treated with 125 µM glutamate (GLU) for 20 min. After 24 h, A) Representative images of nucleus staining by Hoechst and Nuclear area factor (NAF) quantification. Insert: 100X, bar 5 um; panoramic image, 40X, bar: 50 µm. B) GFP from transduced neurons exposed to BACE1 shRNA-miR and SCD1shRNA. 60X, White bars insert: 15 um, panoramic image, 20X, white bar: 50 µm. Relative Units = RU, n= 4, and each experiment was performed in duplicate. All values were normalized to the control neurons. * p < 0.05

**Supplementary Figure 2. General changes of total concentration of PLs from the hippocampus and CSF of cognitive improvement by animal model silenced for BACE1 and SCD1 genes.** The lipid class profiles are expressed as %mol composition observed in the A) hippocampus and B) CSF. All lipid species were measured (means), and the error bars represent the SEM. Asterisk mean: *p< 0.05 between compared groups, according to the color bar. ANOVA or Kruskall-Wallis test followed by Tukey or Dunnet’s T3 post hoc according to the Normality test. Data are expressed as %mol. n=5 per group. The variables in the analyses are as follows: sham ScrBACE1-scrSCD1, sham shBACE1- scrSCD1, sham scrBACE1-shSCD1, sham shBACE1-shSCD1; Isch ScrBACE1- scrSCD1, Isch shBACE1- scrSCD1, Isch scrBACE1- shSCD1, Isch shBACE1- shSCD1.

**Supplementary Figure 3.** **Lipid profile from PCA of the hippocampus, CSF, and serum from cognitive improvement of animal model silenced for BACE1 and SCD1 genes.** Principal components analysis (PCA) lipid profiles from the all groups and comparing Isch group and Isch shBACE1- shSCD1 respectively A-B) hippocampus, C-D) CSF, and F-G) serum. Rho index values in a table per item. The variables in the analyses are as follows: Sham ScrBACE1-scrSCD1, Sham shBACE1-scrSCD1, Sham scrBACE1-shSCD1, Sham shBACE1-shSCD1; Isch ScrBACE1-scrSCD1, Isch shBACE1-scrSCD1, Isch scrBACE1-shSCD1, Isch shBACE1- shSCD1. Abbreviations: PA, phosphatidic acid; PC, phosphatidylcholine; LPC, lysophosphatidylcholine; ePC, etherphosphatidylcholine; PS, phosphatidylserine; ePS, etherphosphatidylserine; PE, phosphatidylethanolamine; LPE, lysophosphatidylethanolamine; ePE, etherphosphatidylethanolamine; PI, phosphatidylinositol; PG, phosphatidylglycerol; SM, sphingomyelin, n=5 per group.

**Supplementary Figure 4. Astrocytes inhibited for BACE1-SCD1 and co-cultured with neurons exposed to glutamate stress.** Representative images of labels for Hoechst (blue), GFAP (red) and SCD1 (green). (A) Astrocytes cocultured with neurons inhibited for BACE1-SCD1 without glutamate (A') Fluorescence profile of SCD1. (B) Astrocytes cocultured with neurons inhibited for BACE1-SCD1 with 125 µM glutamate. (B') Fluorescence profile of SCD1. (C) % of LDH released into the medium of the astrocyte-neuron cocultures. (D) Percentage of condensed nuclei. (E and F) Fluorescence intensity (IF) for GFAP and SCD1. n=4 per replicate. Zoom= 60X. * p<0.05 ** p<0.01. The color of the asterisk over the bar indicates the group with which there is a statistically significant difference. Data are presented as the mean ± SEM.

**Supplemetary Figure 5. Neurons exposed to glutamate stress, co-cultured with astrocytes inhibited for BACE1 and SCD1 exposed**. Representative images of labels for Hoechst (blue), Phalloidin (red) and SCD1 (green). (A) Neurons cocultured with astrocytes inhibited for BACE1-SCD1 without glutamate. (A') Fluorescence profile of SCD1. (B) Neurons cocultured with astrocytes inhibited for BACE1-SCD1 with 125 µM glutamate. (B') Fluorescence profile of SCD1. (C) Percentage of condensed nuclei. (D) Fluorescence intensity (IF) for SCD1. (E) Color convention for the different treatment groups. n=4 per replicate. Zoom= 60X. * p<0.05 ** p<0.01. The color of the asterisk over the bar indicates the group with which there is a statistically significant difference. Data are presented as the mean ± SEM.

**Supplementary Figure 6. Astrocytes inhibited for BACE1-SCD1 and co-cultured with endothelium exposed to glutamate stress.** Representative images of labels for Hoechst (blue), GFAP (red) and SCD1 (green). (A) Astrocytes co-cultured with endothelium inhibited for BACE1-SCD1 without glutamate (A') Fluorescence profile of SCD1. (B) Astrocytes co-cultured with endothelium inhibited for BACE1-SCD1 with 125 µM glutamate. (B') Fluorescence profile of SCD1. (C) % of LDH released into the medium of the co-cultures between astrocytes and endothelium. (D) Percentage of condensed nuclei. (E and F) Fluorescence intensity (IF) for GFAP and SCD1. (G) Color convention for the different treatment groups. n=4 per replicate. Zoom= 60X. * p<0.05 ** p<0.01. The color of the asterisk over the bar indicates the group with which there is a statistically significant difference. Data are presented as the mean ± SEM.

**Supplemtary Figure 7. Endothelium co-cultured with astrocytes inhibited for BACE1-SCD1 and exposed to glutamate stress.** Representative images of labeling for Hoechst (blue), Zonula Occludens-ZO (red) and SCD1 (green). (A) Endothelium co-cultured with astrocytes inhibited for BACE1-SCD1 without glutamate (A') Fluorescence profile of SCD1. (B) Endothelium co-cultured with astrocytes inhibited for BACE1-SCD1 with 125 µM glutamate. (B') Fluorescence profile of SCD1. (C) % of condensed nuclei of endothelial cells. (D) Fluorescence intensity (IF) for SCD1. n=4 per replicate. Zoom= 60X. * p<0.05 ** p<0.01. The color of the asterisk over the bar indicates the group with which there is a statistically significant difference. Data are presented as the mean ± SEM.

**Supplementary Figure 8. Molar percentage of phospholipid families in astrocytes, neurons, and endothelium.** (A) Mole % of each phospholipid family in astrocytes cocultured with neurons, LPC 22:5 and PC 44:10 by treatment group in astrocytes cocultured with neurons. (B) Mole % of each phospholipid family in neurons cocultured with astrocytes. (C) mole % of each phospholipid family in endothelium cocultured with astrocytes. (D) Ratio between LPC and PC in astrocytes cocultured with neurons. (E) Ratio between LPC and PC in neurons cocultured with astrocytes. (F) Ratio between LPC and PC in endothelium cocultured with astrocytes. (n=4) * p<0.05. Data is presented as the mean ± SEM.
