# Supplementary material for: BACE1 and SCD1 are associated with neurodegeneration
Source: Front Aging Neurosci. 2023 Sep 8;15:1194203. doi: 10.3389/fnagi.2023.1194203 (PMC10516302; doi:10.3389/fnagi.2023.1194203)
Supplement: Supplementary file 2 [file Table_1.DOCX]

| **Condition** | **Gender** | **Onset Age** | **Age of death** | **CERAD** | **Braak** | **Thal** |
| --- | --- | --- | --- | --- | --- | --- |
| Control -Healthy | F | NA | 67 | 0 | 1 | 0 |
| Control -Healthy | F | NA | 75 | 0 | 0 | 0 |
| Control -Healthy | M | NA | 69 | A | 1 | 2 |
| Control -Healthy | M | NA | 61 | 0 | 0 | 0 |
| Control -Healthy | F | NA | 44 | 0 | 0 | 0 |
| FAD (E280A) | F | 49 | 62 | B | 4 | 5 |
| FAD (E280A) | F | 44 | 50 | C | 6 | 5 |
| FAD (E280A) | F | 50 | 63 | C | 6 | 5 |
| FAD (E280A) | M | 49 | 59 | C | 6 | 5 |
| FAD (E280A) | F | 51 | 65 | B | 5 | 5 |
| Late SAD | F | 82 | 92 | C | 5 | 4 |
| Late SAD | F | 62 | 74 | B | 5 | 4 |
| Early SAD | F | 55 | 76 | B | 4 | 5 |
| SAD | F | 81 | 94 | B | 4 | 5 |
| SAD | F | 92 | 98 | A | 3 | 3 |
| CADASIL | F | 52 | 65 | B | 1 | 2 |
| CADASIL | F | 35 | 45 | 0 | 0 | 0 |
| CADASIL | F | 32 | 49 | 0 | 0 | 0 |
| CADASIL | M | 41 | 59 | 0 | 0 | 0 |
| CADASIL | F | 55 | 78 | 0 | 0 | 0 |

**Table 1**. Cases used in this study. NA: Non apply
