# Supplementary figures and images for: BACE1 and SCD1 are associated with neurodegeneration
Source: Front Aging Neurosci. 2023 Sep 8;15:1194203. doi: 10.3389/fnagi.2023.1194203 (PMC10516302; doi:10.3389/fnagi.2023.1194203)

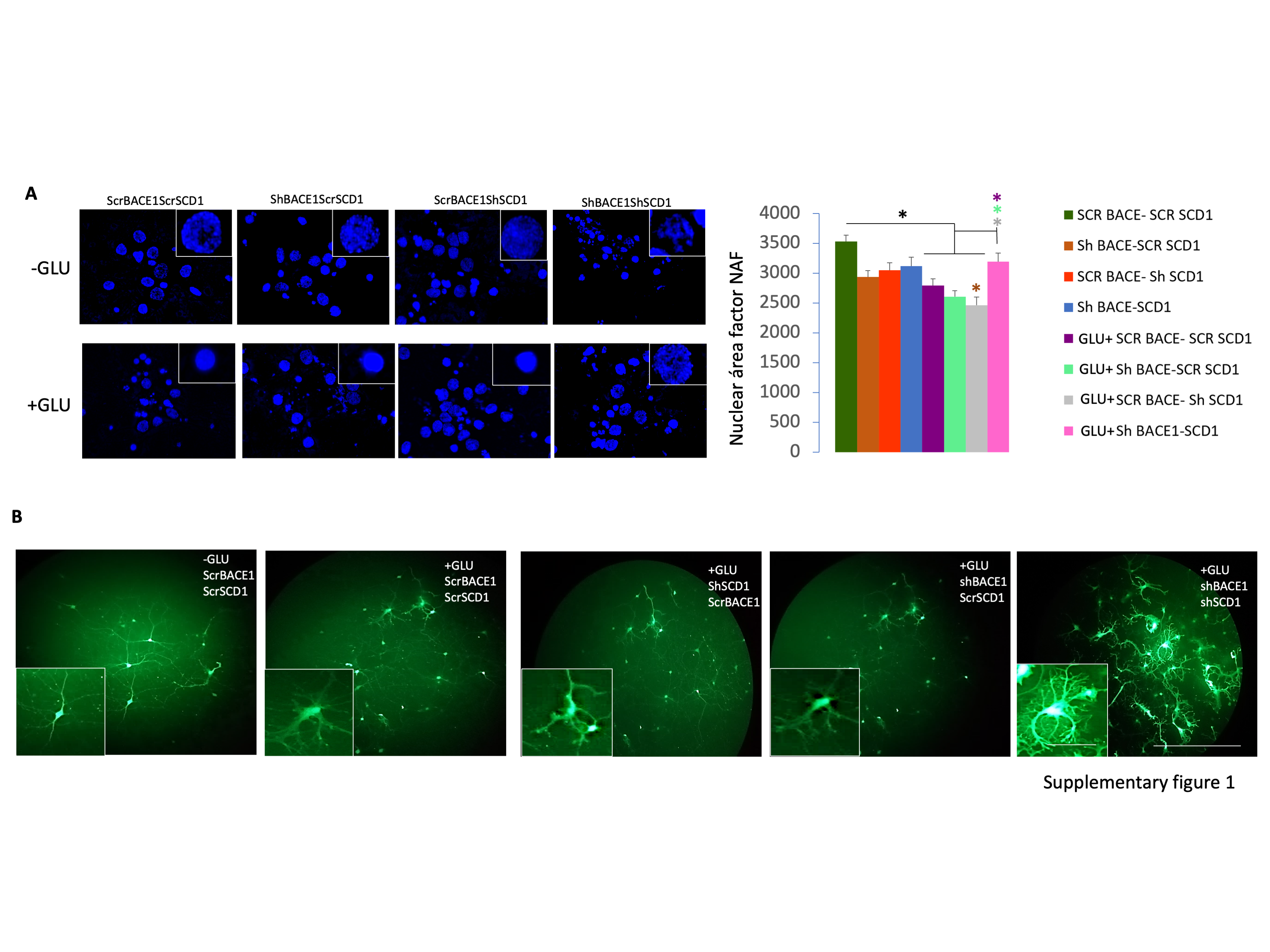

Supplement: Supplementary file 3 [file Image_1.JPEG]

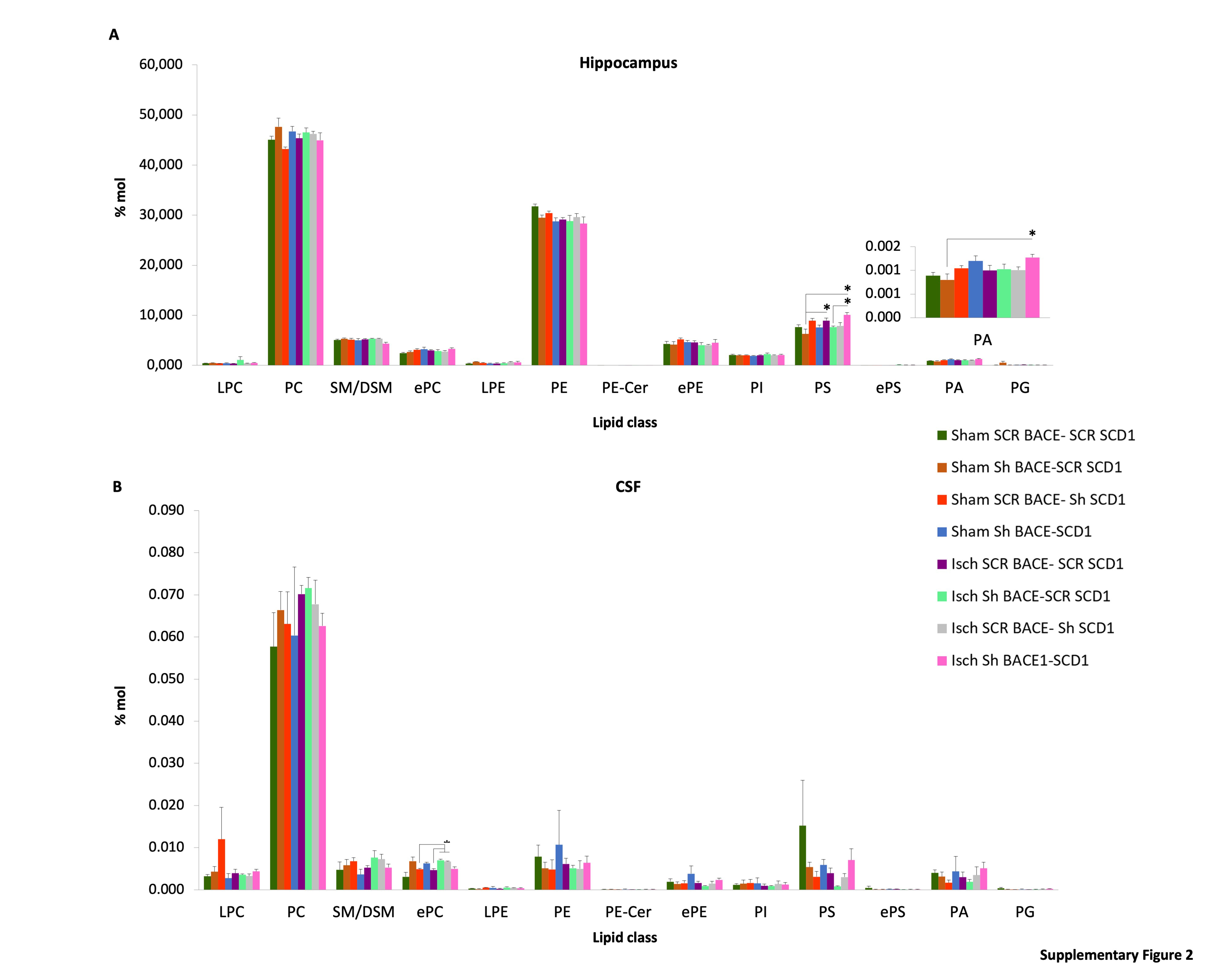

Supplement: Supplementary file 4 [file Image_2.JPEG]

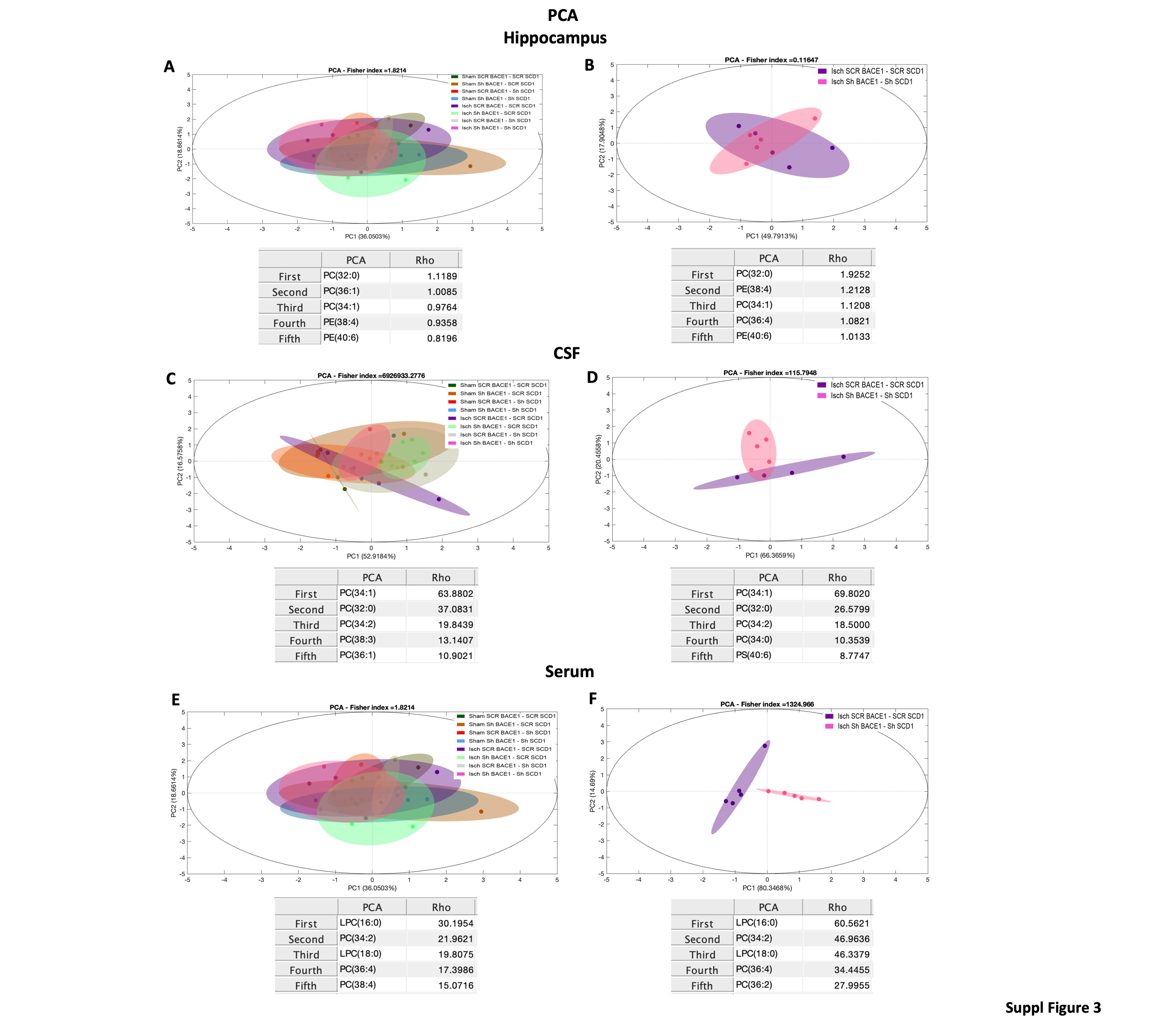

Supplement: Supplementary file 5 [file Image_3.JPEG]

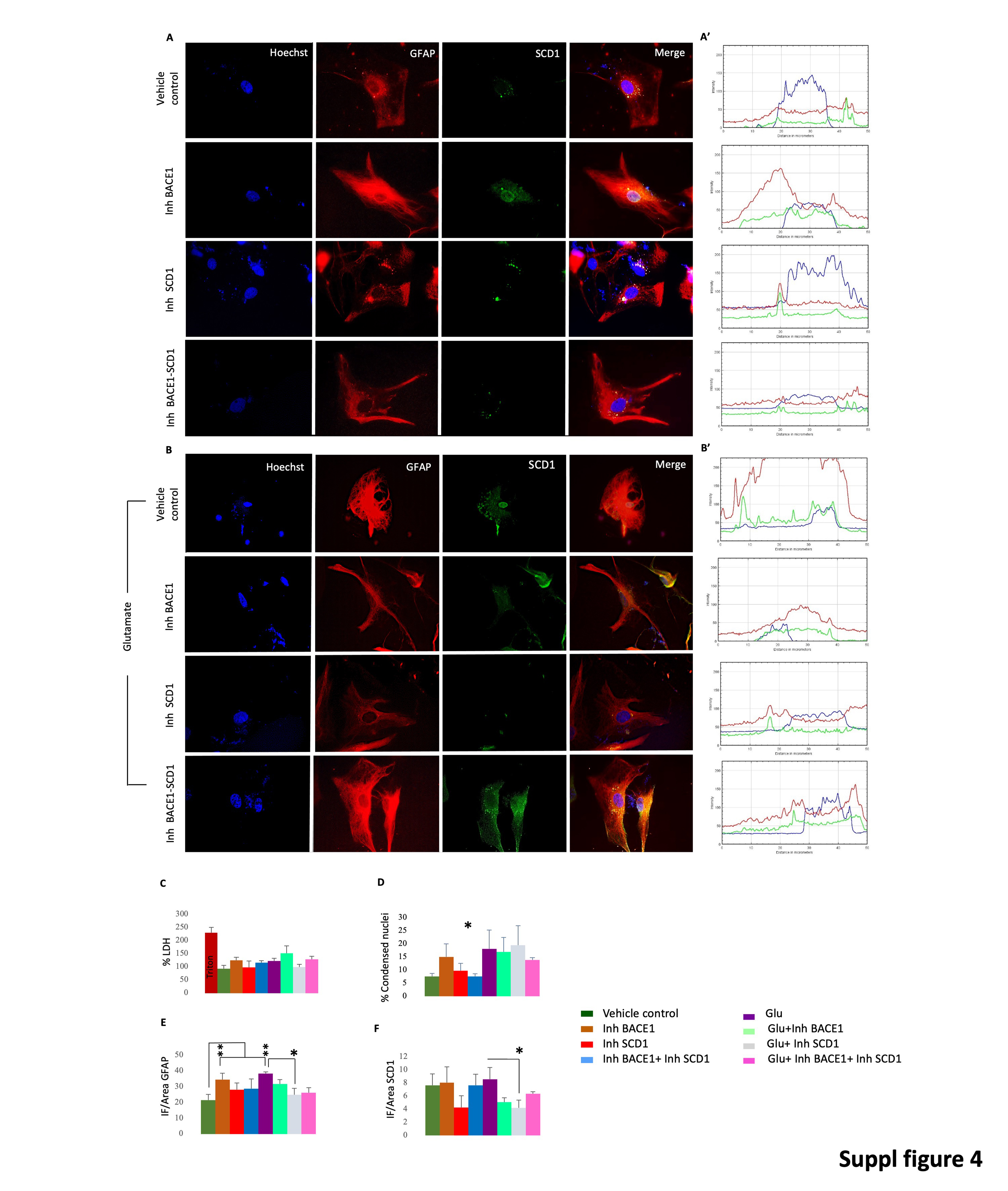

Supplement: Supplementary file 6 [file Image_4.JPEG]

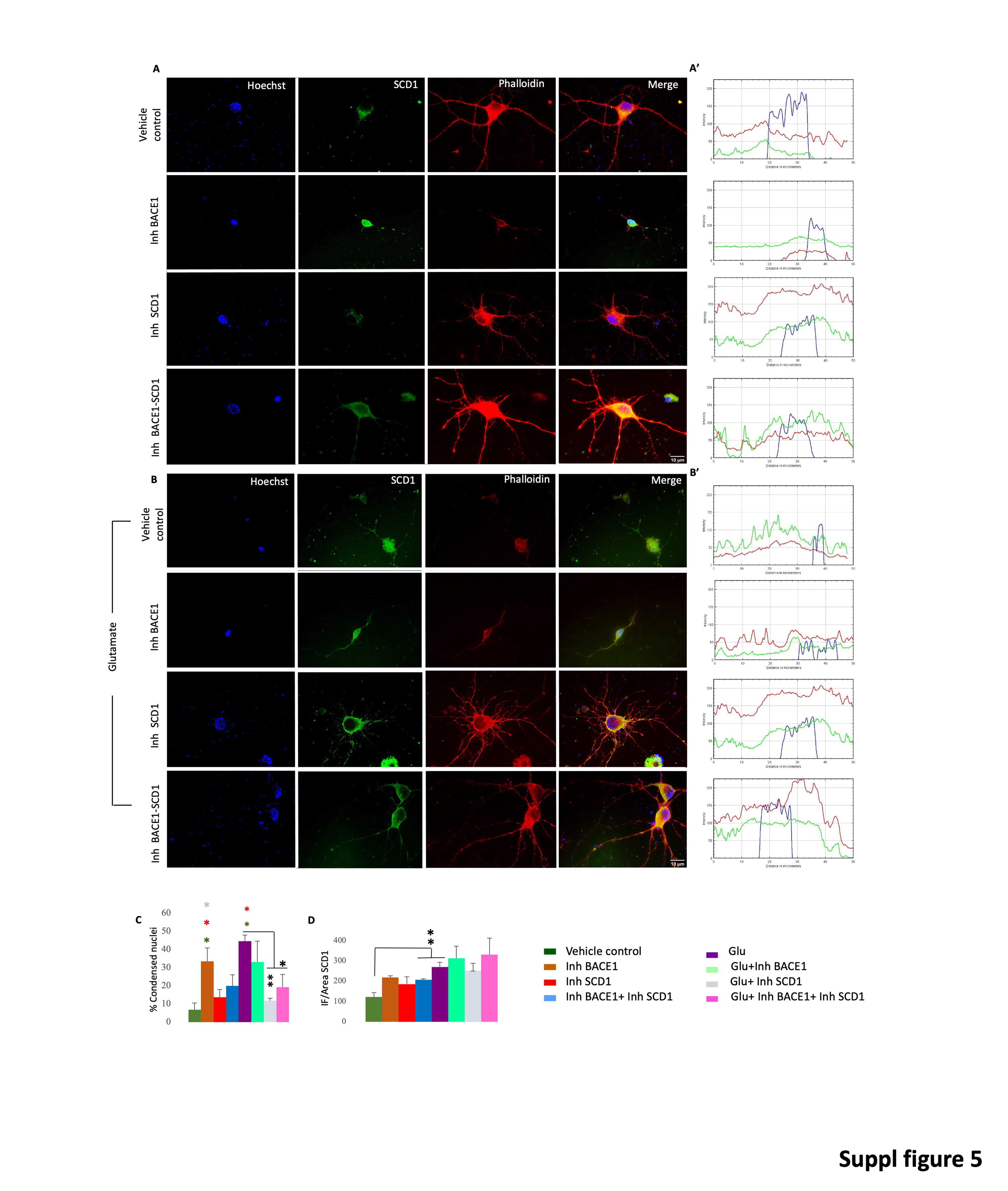

Supplement: Supplementary file 7 [file Image_5.JPEG]

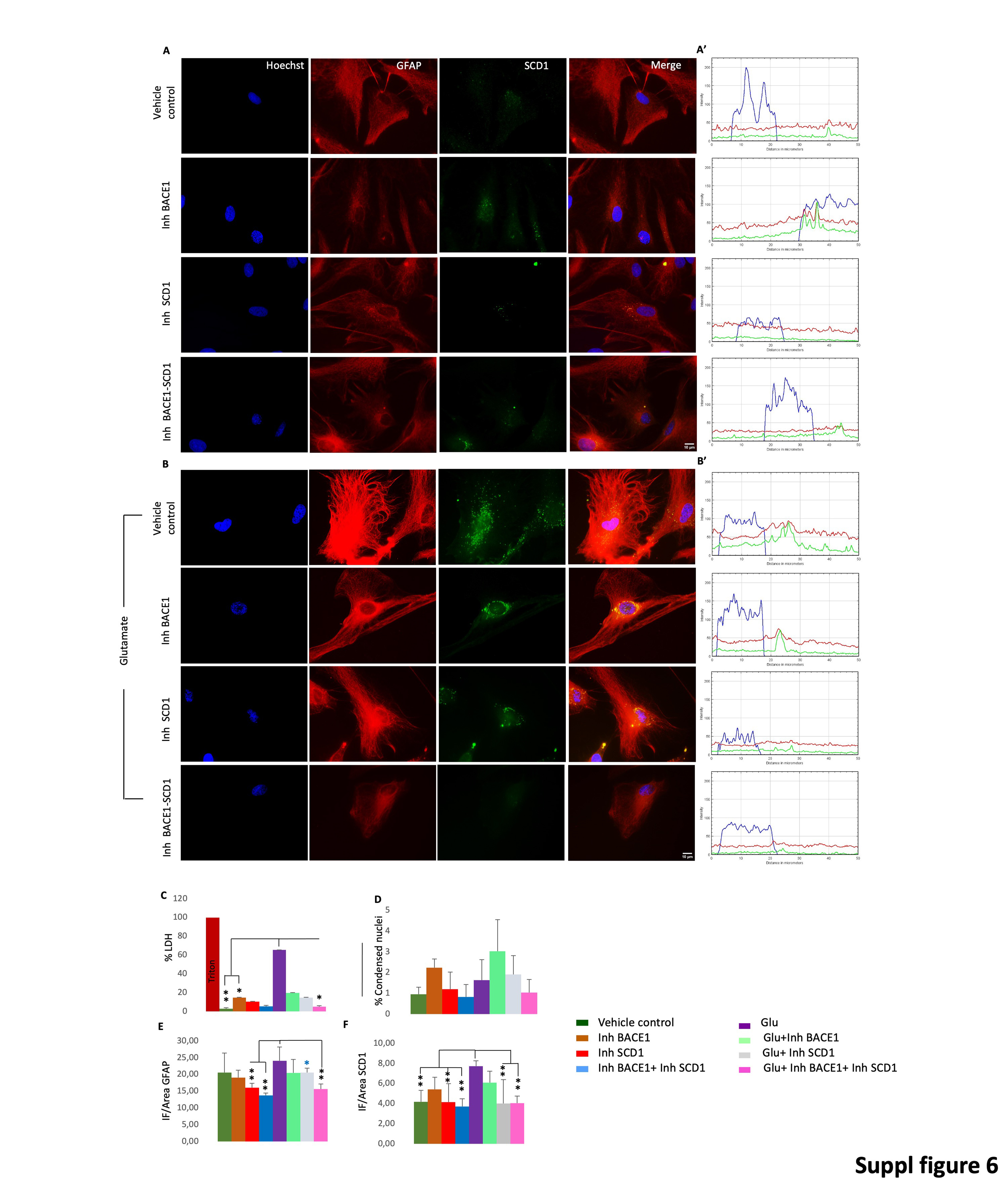

Supplement: Supplementary file 8 [file Image_6.jpeg]

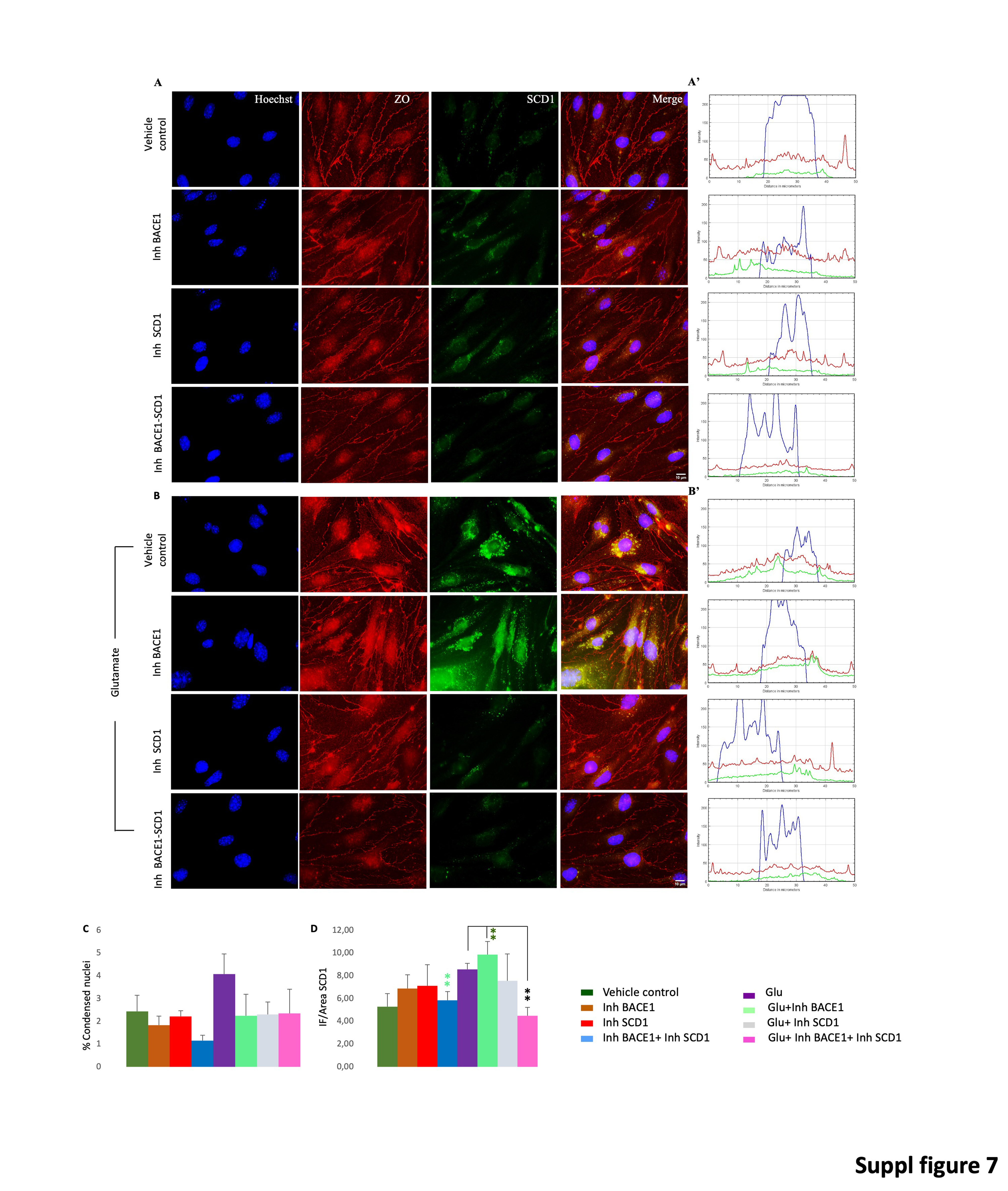

Supplement: Supplementary file 9 [file Image_7.jpeg]
